# Application of machine learning and natural language processing for predicting stroke-associated pneumonia

**DOI:** 10.3389/fpubh.2022.1009164

**Published:** 2022-09-29

**Authors:** Hui-Chu Tsai, Cheng-Yang Hsieh, Sheng-Feng Sung

**Affiliations:** ^1^Department of Radiology, Ditmanson Medical Foundation Chia-Yi Christian Hospital, Chiayi, Taiwan; ^2^Department of Neurology, Tainan Sin Lau Hospital, Tainan, Taiwan; ^3^School of Pharmacy, Institute of Clinical Pharmacy and Pharmaceutical Sciences, College of Medicine, National Cheng Kung University, Tainan, Taiwan; ^4^Division of Neurology, Department of Internal Medicine, Ditmanson Medical Foundation Chia-Yi Christian Hospital, Chiayi, Taiwan; ^5^Department of Nursing, Min-Hwei Junior College of Health Care Management, Tainan, Taiwan

**Keywords:** machine learning, natural language processing, pneumonia, prediction, risk score, stroke

## Abstract

**Background:**

Identifying patients at high risk of stroke-associated pneumonia (SAP) may permit targeting potential interventions to reduce its incidence. We aimed to explore the functionality of machine learning (ML) and natural language processing techniques on structured data and unstructured clinical text to predict SAP by comparing it to conventional risk scores.

**Methods:**

Linked data between a hospital stroke registry and a deidentified research-based database including electronic health records and administrative claims data was used. Natural language processing was applied to extract textual features from clinical notes. The random forest algorithm was used to build ML models. The predictive performance of ML models was compared with the A^2^DS^2^, ISAN, PNA, and ACDD^4^ scores using the area under the receiver operating characteristic curve (AUC).

**Results:**

Among 5,913 acute stroke patients hospitalized between Oct 2010 and Sep 2021, 450 (7.6%) developed SAP within the first 7 days after stroke onset. The ML model based on both textual features and structured variables had the highest AUC [0.840, 95% confidence interval (CI) 0.806–0.875], significantly higher than those of the ML model based on structured variables alone (0.828, 95% CI 0.793–0.863, *P* = 0.040), ACDD^4^ (0.807, 95% CI 0.766–0.849, *P* = 0.041), A^2^DS^2^ (0.803, 95% CI 0.762–0.845, *P* = 0.013), ISAN (0.795, 95% CI 0.752–0.837, *P* = 0.009), and PNA (0.778, 95% CI 0.735–0.822, *P* < 0.001). All models demonstrated adequate calibration except for the A^2^DS^2^ score.

**Conclusions:**

The ML model based on both textural features and structured variables performed better than conventional risk scores in predicting SAP. The workflow used to generate ML prediction models can be disseminated for local adaptation by individual healthcare organizations.

## Introduction

The global burden of stroke is huge and rising (1). According to the most updated statistics from the World Stroke Organization, the global incidence of strokes exceeds 12 million annually and the number of prevalent strokes is more than 100 million worldwide (2). Apart from direct neurological damage, stroke patients are prone to medical complications such as infection (3). Approximately 21 −30% of stroke patients develop post-stroke infections, with pneumonia accounting for a third to half of them (4, 5). Stroke-associated pneumonia (SAP) is not only associated with substantial morbidity and mortality (6–8) but also increases direct healthcare costs (9). Despite the advances in acute stroke treatment over the past decades, the frequency of SAP remains unchanged (4). Effective strategies and interventions are therefore urgently needed to reduce the burden of pneumonia, a potentially preventable complication of stroke.

To prevent SAP, a fundamental first step is the early recognition of high-risk patients, for whom appropriate preventive measures can be taken. Besides, the high-risk patient group is also the main target population for which clinical trials can be designed to test novel interventions for the prevention of pneumonia. Analysis of patient data stored in the Virtual International Stroke Trials Archive showed that most post-stroke pneumonias occurred in the first week and its incidence peaked on the third day after stroke onset (10). Consequently, the risk of developing pneumonia should be assessed as early as possible following stroke. To date, several integer-based risk scores have been developed for predicting SAP (11). Most of the risk models make predictions based on similar predictor variables, such as age, stroke severity, and the presence of dysphagia (11). Hence it is no surprise that these risk models perform comparably regarding discrimination and calibration (11–13). On the other hand, almost all existing SAP prediction models were developed using logistic regression analysis, thus ignoring the potential complex interactions between variables.

With the advances in data science and artificial intelligence, data-driven machine learning (ML) approaches have been increasingly used to develop prediction models in the medical domain (14). These approaches have also been introduced to develop SAP prediction models (15, 16). Compared to conventional parametric techniques like logistic regression, ML approaches have several advantages such as the capability of dealing with high-dimensional data and modeling complex and non-linear relations between data. Furthermore, the ubiquitous adoption of electronic health record (EHR) systems provides an opportunity to use various types of structured and unstructured data for data-driven prediction of clinical outcomes (17–19). Using natural language processing techniques, information extracted from unstructured clinical text has the potential to improve the performance of clinical prediction models (20, 21). Inspired by these ideas, we aimed to explore the value of combining both structured and unstructured textual data in developing ML models to predict SAP.

## Materials and methods

### Data sources

The data sources for this study were the hospital stroke registry and the Ditmanson Research Database (DRD), a deidentified database comprising both administrative claims data and EHRs for research purposes. Supplementary Table 1 lists the general specifics of the data sources. The DRD currently holds clinical information of over 1.4 million patients, including 0.6 million inpatient and 21.5 million outpatient records. It includes both structured data (demographics, vital signs, diagnoses, prescriptions, procedures, and laboratory results) and unstructured textual data (physician notes, nursing notes, laboratory reports, radiology reports, and pathology reports). The hospital stroke registry has prospectively registered all consecutive hospitalized stroke patients since 2007 conforming to the design of Taiwan Stroke Registry (22). Currently, it has enrolled over 12,000 patients. The stroke registry consists of structured data only. Stroke severity was assessed using the National Institutes of Health Stroke Scale (NIHSS) while functional status was evaluated using the modified Rankin Scale (mRS). Information regarding patients' demographics, risk factor profiles, treatments and interventions, complications, and outcomes were collected by trained stroke case managers. To create the dataset for this study, the stroke registry was linked to the DRD using a unique encrypted patient identifier. The study protocol was approved by the Ditmanson Medical Foundation Chia-Yi Christian Hospital Institutional Review Board (approval number: 2022060). Study data were maintained with confidentiality to ensure the privacy of all participants.

### Study population

The derivation of the study population is shown in Supplementary Figure 1. The stroke registry was queried for all stroke hospitalizations, including both acute ischemic stroke (AIS) and intracerebral hemorrhage (ICH), between Oct 2010 and Sep 2021. Only the first hospitalization was considered for each patient. Patients who suffered an in-hospital stroke or already had pneumonia on admission and those whose records could not be linked were excluded. Patients with missing data that made the calculation of pneumonia risk scores impossible were excluded. The study population was randomly split into a training set that consisted of 75% of the patients and a holdout test set comprising the remaining 25% of the patients.

### Predictor and outcome variables

The outcome variable was SAP occurring within the first 7 days after stroke onset (23). As per the protocol of the Taiwan Stroke Registry (22), the diagnosis of SAP was made according to the modified Centers for Disease Control and Prevention criteria (23). Because risk stratification at an early stage after stroke is preferred so that appropriate interventions can be applied, only information available within 24 h of admission was considered. Candidate predictors comprised demographics, pre-stroke dependency (defined as an mRS score of ≥3), risk factors and comorbidities, prior use of medications, physiological measurements, neurological assessment (NIHSS, Glasgow coma scale, and bedside dysphagia screening), as well as routine blood tests (Supplementary Table 2). For predictors that had multiple measurements after admission, such as physiological measurements, neurological assessment, and routine blood tests, only the first measurement was used. Missing values for continuous variables were imputed using the mean of non-missing values. Then each continuous variable was rescaled to a mean of zero and a standard deviation of one.

In the study hospital, admission notes are written in English. To extract predictor features from clinical text, we experimented with three approaches of text representation: a simple “bag-of-words” (BOW) approach, a fastText embedding approach (24), and a deep learning approach using the bidirectional encoder representations from transformers (BERT) (25).

The free text from the History of Present Illness (HPI) section of the admission note was preprocessed through the following steps: spell checking, abbreviation expansion, removal of non-word symbols, lowercase conversion, lemmatization, marking of negated words with the suffix “_NEG” using the Natural Language Toolkit mark_negation function with default parameters (https://www.nltk.org/_modules/nltk/sentiment/util.html), and stop-word removal. Lemmatization, negation marking, and stop-word removal were not needed for the BERT approach.

Supplementary Figure 2 shows an example of feature extraction and preprocessing using the BOW approach. Having no prior knowledge of what information the text can provide, we used an “open-vocabulary” approach (26) to detect features predictive of SAP. We built a document-term matrix where each column represents each unique feature (word or phrase) from the text corpus while the rows represent each patient's clinical document. The preprocessed text was vectorized using the BOW approach with three different types of feature representation (27). In other words, the cells of the document-term matrix represent the counts of each word within each document (term frequency), the absence or presence of each word within each document (binary representation), or the term frequency with inverse document frequency weighting, respectively. Because medical terms are commonly comprised of two words or even more, we also experimented with adding word bigram features (two-word phrases) to the basic BOW model. To reduce noise such as redundant and less informative features as well as to improve training efficiency (28), feature selection was performed by selecting the top 20 words or phrases that appeared in the documents of patients with SAP and those without based on chi-square statistics (29). Supplementary Figures 3–6 show the top 20 selected words or phrases for each feature representation method.

The fastText subword embedding model is an extension of Word2Vec, which uses skip-gram model to represent each word in the form of character n-grams (24). It allows handling out-of-vocabulary words in the training samples. We resumed training of the model from a pre-trained model called BioWordVec using the training set. Then the clinical text was vectorized using the trained model. BioWordVec was originally created from unlabeled biomedical text from PubMed and Medical Subject Headings using the fastText subword embedding model (30). Later, the original BioWrodVec was extended by adding the Medical Information Mart for Intensive Care III clinical notes to the training text corpus (31).

The BERT model is a contextualized word representation model, which allows modeling long-distance dependencies in text. The BERT model is pre-trained based on masked language modeling and next sentence prediction using bidirectional transformers on the general Toronto BookCorpus and English Wikipedia corpus (25). For this study, we used a domain-specific BERT model, i.e., ClinicalBERT (32), which was pre-trained on the Medical Information Mart for Intensive Care III clinical notes. We fine-tuned the BERT model using the training set to predict SAP. The text from the training set was preprocessed and split into BERT tokens. Since the BERT model can only accommodate 512 tokens, the input text was truncated to 512 tokens. For BERT fine-tuning, the batch size was set at 16. The learning rate of the Adam optimizer was set at 2 × 10^−5^ and the number of epochs was 3. Then text from the training and test sets was vectorized by averaging all contextualized word embeddings output by the fine-tuned BERT model.

### SAP risk scores

To compare the predictive performance of ML models, four conventional SAP risk scores (Table 1) were used as comparison models based on variables available in the dataset. The total score of each SAP risk score is calculated by summing up the scores of all its items. A higher total score indicates a greater risk of developing SAP. The A^2^DS^2^ score was derived from clinical data of patients with AIS from the Berlin Stroke Register (33). It comprised age (1 point for ≥75), atrial fibrillation (1 point), dysphagia (2 points), male sex (1 point), and NIHSS (3 points for 5–15 and 5 points for ≥16). The 22-point ISAN score was developed using data of patients with AIS or ICH from a national United Kingdom registry (34). It consisted of pre-stroke dependency (2 points), male sex (1 point), age (3 points for 60–69, 4 points for 70–79, 6 points for 80–89, and 8 points for ≥90), and NIHSS (5 points for 5–15, 8 points for 16–20, and 10 points for ≥21). The PNA score, created using data of AIS patients from a single academic institution, included age (1 point for ≥70), history of diabetes (1 point), and NIHSS (3 points for 5–15 and 5 points for >15) (35). The ACDD^4^ score, developed based on a single-site cohort of patients with AIS or ICH, was composed by age (1 point for ≥75), congestive heart failure (1 point), dysarthria (1 point), and dysphagia (4 point) (36).

**Table 1:** Risk scores for predicting stroke-associated pneumonia.

	**A^2^DS^2^**	**ISAN**	**PNA**	**ACDD^4^**
**Age**	
≥70			+1	
≥75	+1			+1
60–69		+3		
70–79		+4		
80–89		+6		
≥90		+8		
Male	+1	+1		
Diabetes			+1	
AF	+1			
CHF				+1
Pre-stroke dependency		+2		
**NIHSS**	
5–15	+3	+5	+3	
≥16	+5		+5	
16–20		+8		
≥21		+10		
Dysphagia	+2			+4
Dysarthria				+1

AF, atrial fibrillation; CHF, congestive heart failure; NIHSS, National Institutes of Health Stroke Scale.

### Machine learning models

ML models were constructed based on structured variables, features extracted from the text, or a combination of both (Supplementary Figure 7). For comparison of classifier performance, simple logistic regression was used as the baseline. Because the performance of ML classifiers can be affected by class imbalance, we experimented with both oversampling and under-sampling methods to maintain the ratio of majority and minority classes as 1:1, 2:1, or 3:1 (37). The random forest (RF) algorithm was used to build classifiers. RF is a classifier ensemble method that consists of a set of decision tree classifiers. During the learning process, RF iteratively adopts the bootstrap aggregating method to select samples and randomly selects a subset of predictors. In each iteration, each set of bootstrap samples with a subset of predictors is used to generate a decision tree. In the end, the algorithm outputs a whole forest of decision trees, which can be used for prediction by a majority vote of the trees.

During the training process (Supplementary Figure 7), we first experimented with different combinations of text vectorization techniques and resampling methods without hyperparameter tuning. We repeated 10-fold cross-validation 10 times to estimate the performance of classifiers. The best combination of text vectorization and resampling methods was determined based on the area under the receiver operating characteristic curve (AUC). Next, for each text vectorization technique with its corresponding best resampling method, we trained classifiers with hyperparameter tuning using 10 times of 10-fold cross-validation to determine the best number of decision trees in the random forest. Then we trained the final ML models from the whole training set using the best hyperparameter. The generated ML models were tested on the holdout test set. Shapley additive explanations (38) was used to interpret the model output. The experiments were carried out by using scikit-learn, imbalanced-learn, gensim, transformers, sentence-transformers, and SHAP libraries within Python 3.7 environment.

### Statistical analysis

Categorical variables were presented with counts and percentages. Continuous variables were reported as medians and interquartile ranges. Differences between groups were tested by Chi-square tests for categorical variables and Mann-Whitney *U* tests for continuous variables.

Because accuracy may not be appropriate for model evaluation under imbalanced scenarios (39), the AUC was chosen as the primary evaluation metric for comparing the performance of prediction models on the holdout test set. The AUC for SAP risk scores was calculated using the receiver operating characteristic (ROC) analysis to determine the ability of each risk score to predict SAP. The method for ROC analysis was detailed in the Supplementary Methods in the Supplementary material. AUCs were calculated and compared using DeLong's method (40). The AUC ranges from 0 to 1, with 0.5 indicating random guess and 1 indicating perfect model discrimination. A model with an AUC value above 0.7 is considered acceptable for clinical use (41). The point closest to the upper left corner of the ROC curve (42), which represents the optimal trade-off between sensitivity and specificity, was considered the cut-off value for each SAP score. Then each SAP score was transformed into a binary variable for calculating accuracy, precision (positive predictive value), recall (sensitivity), and F1 score. Model calibration was evaluated by the Hosmer-Lemeshow test and visualized by the calibration plot (43), which depicts the observed risk vs. the predicted risk.

All statistical analyses were performed using Stata 15.1 (StataCorp, College Station, Texas) and R version 4.1.1 (R Foundation for Statistical Computing, Vienna, Austria). Two-tailed *P* values of 0.05 were considered significant.

## Results

### Characteristics of the study population

The study population consisted of 5,913 patients including 4,947 (83.7%) with AIS and 966 (16.3%) with ICH. A total of 450 (7.6%) patients developed SAP. Table 2 lists their baseline characteristics. Patients with SAP were older, more likely to be male, and more likely to have atrial fibrillation, congestive heart failure, pre-stroke dependency, dysarthria, and dysphagia, but less likely to have hyperlipidemia. They had a higher pre-stroke mRS, NIHSS, and white blood cell (WBC) count as well as a lower consciousness level than those without SAP. The training set consisted of 4,434 patients and the remaining 1,479 patients comprised the holdout test set (Supplementary Table 3).

**Table 2:** Baseline characteristics of the study population.

**Characteristic**	**Total (*N* = 5,913)**	**SAP (*N* = 450)**	**No SAP (*N* = 5,463)**	***P* †**
Age	70 (59–78)	72 (61–80)	69 (59–78)	<0.001
Male	3,643 (61.6)	308 (68.4)	3,335 (61.0)	0.002
Hypertension	4,739 (80.2)	361 (80.2)	4,378 (80.1)	0.966
Diabetes	2,422 (41.0)	188 (41.8)	2,234 (40.9)	0.714
Hyperlipidemia	3,167 (53.6)	187 (41.6)	2,980 (54.6)	<0.001
AF	822 (13.9)	106 (23.6)	716 (13.1)	<0.001
CHF	226 (3.8)	30 (6.7)	196 (3.6)	0.001
COPD	397 (6.7)	34 (7.6)	363 (6.6)	0.458
Smoking	2,431 (41.1)	202 (44.9)	2,229 (40.8)	0.090
Pre-stroke dependency	562 (9.5)	80 (17.8)	482 (8.8)	<0.001
Pre-stroke mRS	0 (0–0)	0 (0–1)	0 (0–0)	<0.001
NIHSS	5 (3–11)	17 (9–27)	5 (3–10)	<0.001
GCS	15 (14–15)	13 (8–15)	15 (15–15)	<0.001
Dysphagia	1,195 (20.2)	282 (62.7)	913 (16.7)	<0.001
Dysarthria	3,039 (51.4)	338 (75.1)	2,701 (49.4)	<0.001
Glucose (mmol/L)	7.38 (6.11–9.99)	7.77 (6.27–10.43)	7.33 (6.11–9.96)	0.030
WBC (10^9^/L)	7.68 (6.19–9.61)	8.49 (6.63–10.96)	7.63 (6.16–9.47)	<0.001
A^2^DS^2^	4 (1–5)	6 (4–6)	3 (1–5)	<0.001
ISAN	7 (4–10)	11 (8–14)	7 (4–9)	<0.001
PNA	4 (1–5)	5 (4–6)	4 (1–5)	<0.001
ACDD^4^	1 (0–2)	5 (2–5)	1 (0–2)	<0.001

† P values are comparisons between patients with SAP and those without SAP for each variable.

Data are given as n (%) and median (interquartile range).

AF, atrial fibrillation; CHF, congestive heart failure; COPD, chronic obstructive pulmonary disease; GCS, Glasgow coma scale; mRS, modified Rankin Scale; NIHSS, National Institutes of Health Stroke Scale; SAP, stroke-associated pneumonia; WBC, white blood cells.

### Construction of ML models

Supplementary Figure 8 shows the estimates of AUC obtained from 10 times of 10-fold cross-validation in the training set. In general, the RF algorithm outperformed logistic regression when structured variables or both structured and textual features were used to build classifiers. By contrast, logistic regression models had higher AUCs than RF classifiers when only textual features were used. Resampling methods generally improved the performance of ML classifiers. Overall, RF classifiers based on both structured variables and textual features attained higher AUCs than the other classifiers. Text representation using the BOW approach performed better than that using the fastText embedding or BERT approach. The highest AUC was achieved by the ML model using the combination of text vectorization with BOW (binary representation) and 1:2 under-sampling of data.

Supplementary Table 4 shows the performance of ML models on the holdout test set and the number of decision trees used to build the RF classifiers. Supplementary Table 5 lists *P* values for pairwise comparisons of AUCs between these models. In general, ML models based on both structured and textual features achieved higher AUCs than those based on textual features alone. The ML model using the combination of text vectorization with BOW (binary representation) also had the highest AUC among all ML models. Therefore, it was chosen as the final model (ML Model A). For comparison with conventional risk scores, the ML model based on structured variables alone (ML Model B) was also evaluated.

### Comparison with conventional risk scores

**Figure 1:**
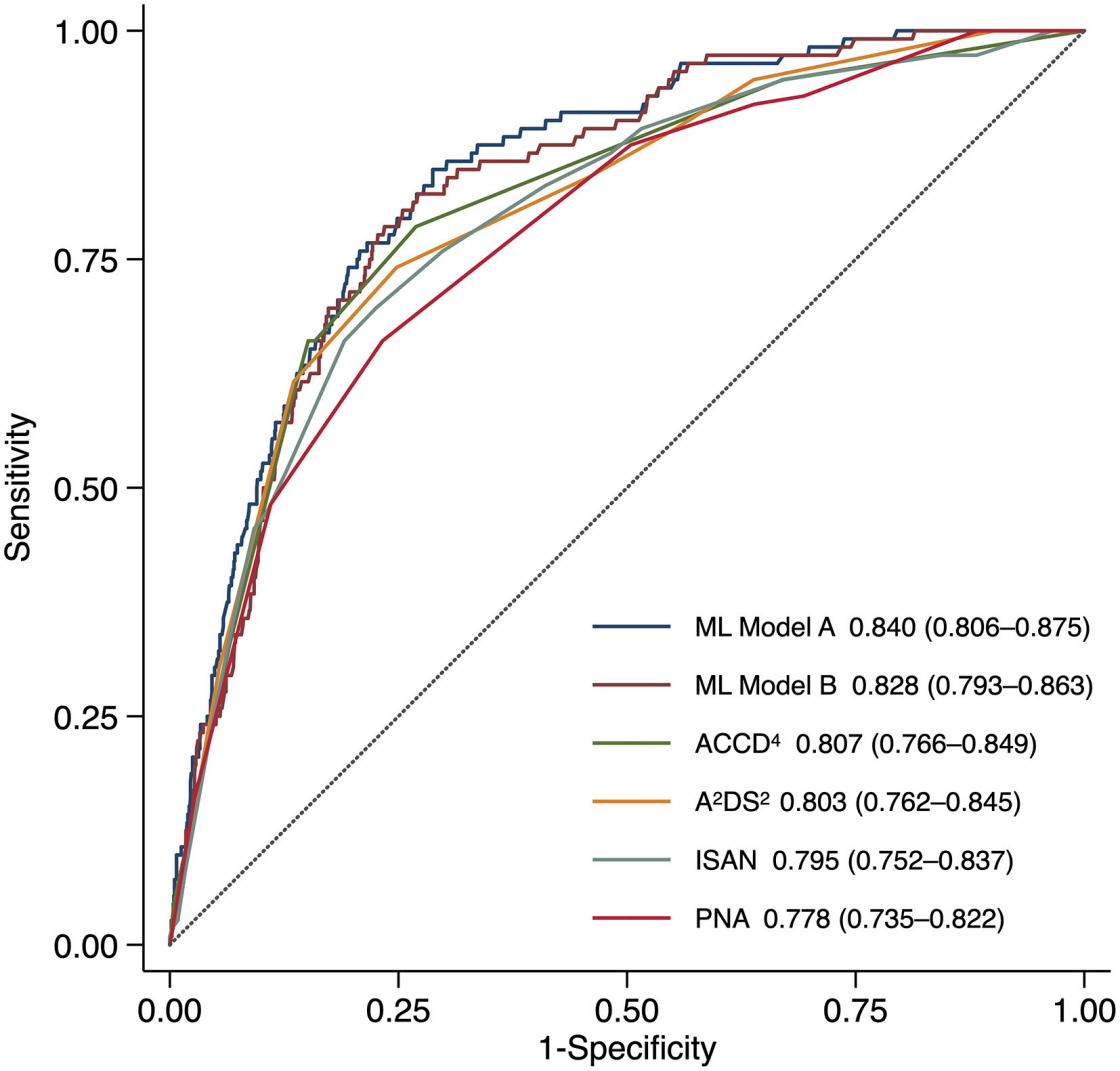
Receiver operating characteristic curves for predicting stroke-associated pneumonia in the holdout test set by existing pneumonia risk scores and two ML models. ML Model A was built using both structured variables and features extracted from the text. ML Model B was built using structured variables alone. The AUC (95% CI) is shown for each model. AUC, area under the receiver operating characteristic curve; CI, confidence interval; ML, machine learning.

**Figure 2:**
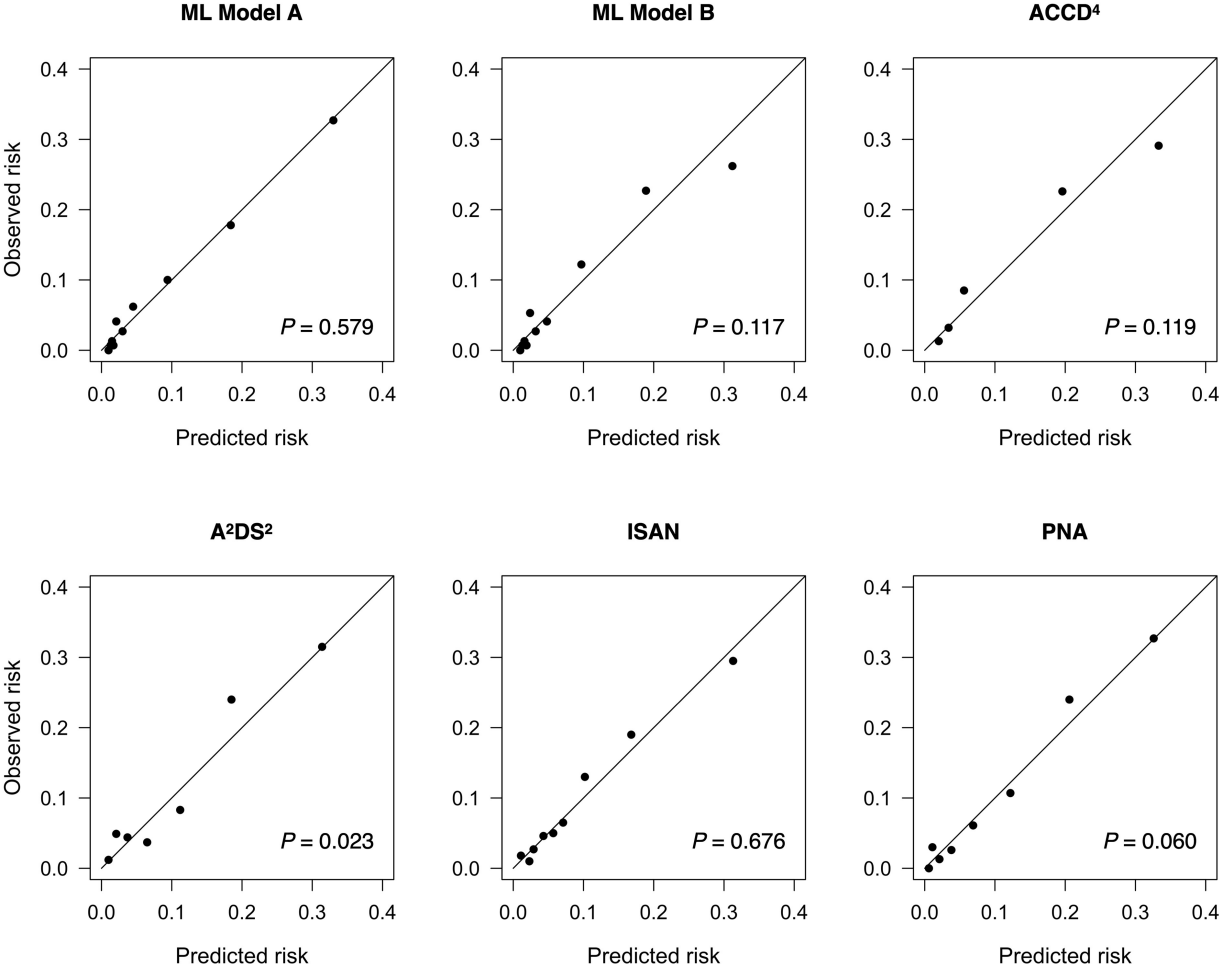
Calibration plots for predicting stroke-associated pneumonia in the holdout test set by existing pneumonia risk scores and two ML models. The *P* value for the Hosmer-Lemeshow test is shown for each model. ML, machine learning.

By determining the point closest to the upper left corner of the ROC curve (42) the cut-off value for predicting SAP was 4.5 points for A^2^DS^2^, 9.5 points for ISAN, 4.5 points for PNA, and 1.5 points for ACDD^4^, respectively. The cut-off value for ML models was set at the probability of 0.5. Accuracy, precision, recall, and F1 score were calculated based on these cut-off values. Table 3 lists the performance of ML models and conventional SAP risk scores on the holdout test set. Among all prediction models, ML Model A attained the highest AUC, accuracy, and F1 score. Figure 1 plots the ROC curves of the four SAP risk scores and two ML models. All the prediction models achieved an AUC value >0.7. ML Model A had the highest AUC [0.840, 95% confidence interval (CI) 0.806–0.875], which was significantly higher than those of ML Model B (0.828, 95% CI 0.793–0.863, *P* = 0.040), ACDD^4^ (0.807, 95% CI 0.766–0.849, *P* = 0.041), A^2^DS^2^ (0.803, 95% CI 0.762–0.845, *P* = 0.013), ISAN (0.795, 95% CI 0.752–0.837, *P* = 0.009), and PNA (0.778, 95% CI 0.735–0.822, *P* <0.001). Figure 2 shows the calibration plots and *P* values for the Hosmer-Lemeshow test for the prediction models. ML Model A was well-calibrated over the entire risk range with all points lying close to the 45-degree line (*P* = 0.579). All the other prediction models also demonstrated adequate calibration except for the A^2^DS^2^ score (*P* = 0.023).

**Table 3:** Performance of prediction models for predicting SAP.

**Model**	**AUC (95% CI)**	**Accuracy**	**Precision**	**Recall**	**F1 score**
ML model A	0.840 (0.806–0.875)	83.2%	0.254	0.634	0.363
ML model B	0.828 (0.793–0.863)	76.3%	0.212	0.786	0.334
A^2^DS^2^	0.803 (0.762–0.845)	75.1%	0.197	0.741	0.311
ISAN	0.795 (0.752–0.837)	76.9%	0.202	0.696	0.313
PNA	0.778 (0.735–0.822)	75.9%	0.189	0.661	0.294
ACDD^4^	0.807 (0.766–0.849)	73.5%	0.193	0.786	0.310

AUC, area under the receiver operating characteristic curve; CI, confidence interval; ML, machine learning; SAP, stroke-associated pneumonia.

### Influential features selected by ML models

**Figure 3:**
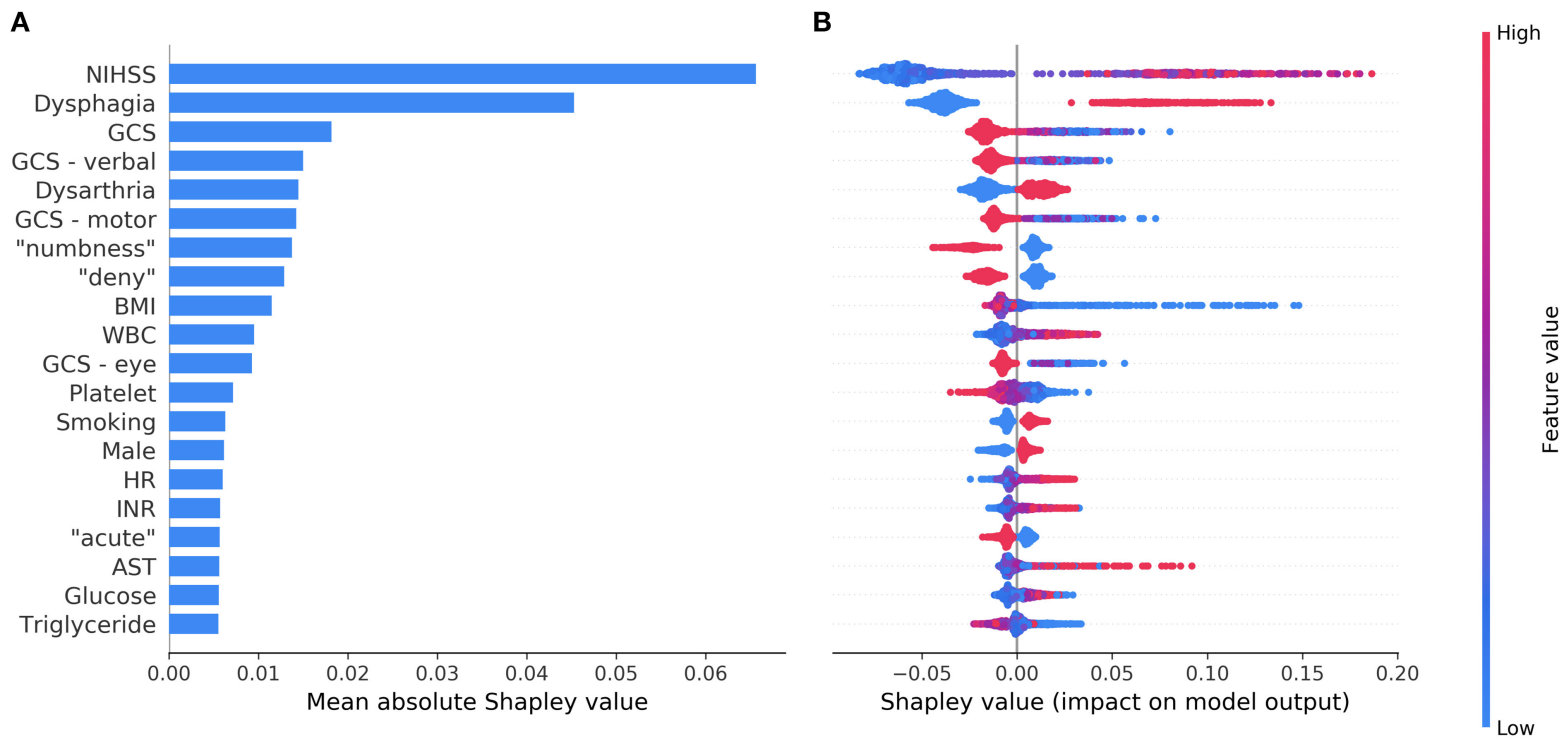
The top 20 most influential features identified by the model based on both structured variables and features extracted from the text. The average impact of each feature on the model output was quantified as mean absolute Shapley values **(A)**. Each feature's individual Shapley values for each patient are depicted in a beeswarm plot **(B)**, where a dot's position on the x-axis denotes each feature's contribution to the model prediction for the corresponding patient. The color of the dot specifies the relative value of the corresponding feature. AST, aspartate aminotransferase; BMI, body mass index; GCS, Glasgow coma scale; HR, heart rate; INR, international normalization ratio; NIHSS, National Institutes of Health Stroke Scale; WBC, white blood cells.

Figure 3A shows the top 20 most influential features selected by ML Model A ordered by the mean absolute Shapley value, which indicates the global importance of each feature on the model output. Figure 3B presents the beeswarm plot depicting the Shapley value for every patient across these features, demonstrating each feature's contribution to the model output. According to the magnitude and direction of the Shapley value, higher values of NIHSS, WBC count, heart rate, blood glucose, international normalization ratio, and aspartate aminotransferase were associated with a higher risk of SAP, while lower values of Glasgow coma scale total score and its component (verbal, motor, and eye) scores, body mass index, platelet count, and triglyceride were associated with a higher risk of SAP. Male patients and those with dysphagia, dysarthria, or current smoking were more likely to have SAP. Among the textual features, the presence of “numbness”, “deny”, or “acute” in the HPI of the admission note was associated with a decreased risk of SAP. The top 20 most influential features selected by ML Model B are shown in Supplementary Figure 9 for reference.

## Discussion

In this exploratory study, the predictive performance of ML models was nominally higher than those using conventional SAP risk scores in terms of discrimination. Notably, the ML model built on both structured and unstructured textual data performed significantly better than the ML model built on structured data alone as well as all the conventional risk scores. Besides, we discovered several influential features or predictors of SAP using Shapley values. These predictors might help early stratification of stroke patients who are more likely to develop SAP.

### Predictors of SAP

Among the top 20 influential predictors selected by the ML model, NIHSS score, Glasgow coma scale score, dysphagia, dysarthria, current smoking, male sex, WBC count, and blood glucose were known predictors of SAP, which have been included in conventional SAP risk scores (11, 33–36). A higher value of international normalized ratio in the context of stroke generally denotes the use of vitamin K antagonist and preexisting atrial fibrillation, which is also a known risk factor for SAP (11, 33). Interestingly, the ML model identified additional predictors, such as lower values of body mass index, platelet count, and triglyceride as well as higher values of heart rate and aspartate aminotransferase. Previous studies have found significantly lower body mass index, platelet count, and triglyceride as well as higher aspartate aminotransferase in stroke patients with SAP than those without (16, 44, 45). All these factors indicate poorer nutritional status, which may have a role in the development of SAP (45). Higher heart rate at rest was associated with poorer functional status in the elderly and predicted subsequent functional decline independently of cardiovascular risk factors (46). Higher initial in-hospital heart rate also predicted poorer stroke outcomes (47). The potential influence of these additional predictors on the development of SAP may warrant further research. We speculate that these factors are missing in conventional SAP risk scores either because logistic regression models cannot handle complex interactions and non-linear relationships among variables, or simply because they were not expected to be predictors of SAP and thus not investigated in previous studies.

### Hidden information from clinical text

The key finding of the present study was that the information extracted from unstructured clinical text could improve the prediction of SAP. However, the reason why the identified textual features (words) were associated with the risk of SAP may not be readily discernible unless these words and their context are examined simultaneously. For example, stroke patients who complain of “numbness” are generally fully conscious and may suffer a pure sensory stroke or sensorimotor stroke due to a small ischemic lesion (48, 49), which carries a low risk of pneumonia. Likewise, patients who can provide a history of their illness and “deny” the presence of certain symptoms are likely to have clear consciousness and may have mild neurological impairment. Furthermore, the mode of symptom onset can influence the pre-hospital delay of stroke patients (50). Patients experiencing “acute” symptoms are generally admitted to the stroke unit earlier while stroke unit care is associated with a lower frequency of SAP (4). These findings demonstrate that useful and informative predictors could be uncovered from unstructured clinical text through natural language processing and ML without human curation.

### Clinical significance and implications

SAP has traditionally been attributed to aspiration secondary to dysphagia, impaired cough reflex, or reduced level of consciousness (3). Nonetheless, up to 40% of SAP may be unrelated to aspiration (8). Other causes such as bacteremia due to dysfunction of the gut immune barrier (51) and stroke-induced immune suppression (3, 52) may also contribute to the development of SAP. So far there is no sufficient evidence from clinical trials to demonstrate the effect of dysphagia screening protocols on the prevention of SAP (53). Meta-analyses of randomized trials have also failed to support the use of preventive antibiotic therapy to decrease the risk of SAP in acute stroke patients (54, 55). Furthermore, only weak evidence exists about whether intensified oral hygiene care reduces the risk of SAP (56, 57). Therefore, it is still a major challenge to find new therapeutic approaches to prevent SAP.

Despite this, adequate stratification of SAP risk is not without value. First, a good understanding of the risk of this serious complication of stroke will improve communication between physicians, patients, and caregivers. Second, the identification of at-risk patient groups allows recruiting suitable patients into clinical trials to test preventive interventions for SAP. Up to two-thirds of SAP occurs in the first week, with a peak incidence on the third day after stroke onset (10). Therefore, early stratification of SAP risk is beneficial in both clinical practice and research settings. The ML model developed in this study, which was based on information available within 24 h of admission, is well–suited for use in this context.

### Limitations

This study has several limitations to be addressed. First, even though data-driven ML modeling has the potential to identify novel predictors, the predictor-outcome relationships discovered from data do not translate into a causal relationship (58). Second, we only extracted textual information from the HPI section of the admission note and did not investigate other clinical notes such as nursing notes and image reports. Further studies may examine the usefulness of information extracted from different kinds of clinical notes. Third, this study used oversampling and under-sampling techniques to solve the problem of data imbalance. Other data preprocessing approaches, such as synthetic minority oversampling technique or its variants (37), can be explored in future studies. Fourth, several criteria exist to determine the most appropriate cut-off value for tests with continuous outcomes (42). The use of different criteria can result in different cut-off values for SAP risk scores, hence different results of accuracy, precision, recall, and F1 score. Fifth, high percentages of missingness for certain potential predictors, such as glycosylated hemoglobin, might prevent the ML algorithm from identifying their significance. Finally, this is a single-site study, and the generalizability of the study findings is limited. For example, the vocabulary and terms used for clinical documentation may differ across healthcare settings. Nevertheless, the procedure of model development can be replicated in individual hospitals to generate customized versions of SAP prediction models.

### Conclusions

We demonstrated that it is feasible to build ML models to predict SAP based on both structured and unstructured textual data. Using natural language processing, pertinent information extracted from clinical text can be applied to improve the performance of SAP prediction models. In addition, ML algorithms identified several novel predictors of SAP. The workflow used to generate these models can be disseminated for local adaptation by individual healthcare organizations.

## Data availability statement

The data analyzed in this study is subject to the following licenses/restrictions. The data used in this study cannot be made available because of restrictions regarding the use of EHR data. Requests to access these datasets should be directed to S-FS, sfusng@cych.org.tw.

## Ethics statement

The studies involving human participants were reviewed and approved by the Ditmanson Medical Foundation Chia-Yi Christian Hospital Institutional Review Board. Written informed consent for participation was not required for this study in accordance with the national legislation and the institutional requirements.

## Author contributions

Study concept and design: H-CT and S-FS. Acquisition of data and study supervision: S-FS. Drafting of the manuscript: H-CT and C-YH. All authors analysis and interpretation of data, critical revision of the manuscript for important intellectual content, and had full access to all the data in the study and take responsibility for the integrity of the data and the accuracy of the data analysis.

## Funding

This research was supported in part by the Ditmanson Medical Foundation Chia-Yi Christian Hospital-National Chung Cheng University Joint Research Program [grant number CYCH-CCU-2022-14]. The funder of the research had no role in the design and conduct of the study, interpretation of the data, or decision to submit for publication.

## Conflict of interest

The authors declare that the research was conducted in the absence of any commercial or financial relationships that could be construed as a potential conflict of interest.

## Publisher's note

All claims expressed in this article are solely those of the authors and do not necessarily represent those of their affiliated organizations, or those of the publisher, the editors and the reviewers. Any product that may be evaluated in this article, or claim that may be made by its manufacturer, is not guaranteed or endorsed by the publisher.

## References

[1] GBD 2019 Stroke Collaborators. Global, regional, and national burden of stroke and its risk factors, 1990–2019: a systematic analysis for the Global Burden of Disease Study 2019. Lancet Neurol. (2021) 20:795–820. 10.1016/S1474-4422(21)00252-0 34487721 PMC8443449

[2] Feigin VL Brainin M Norrving B Martins S Sacco RL Hacke W. World stroke organization (WSO): global stroke fact sheet 2022. Int J Stroke. (2021) 17:18–29. 10.1177/17474930211065917 34986727

[3] Elkind MSV Boehme AK Smith CJ Meisel A Buckwalter MS. Infection as a stroke risk factor and determinant of outcome after stroke. Stroke. (2020) 51:3156–68. 10.1161/STROKEAHA.120.030429 32897811 PMC7530056

[4] Badve MS Zhou Z van de Beek D Anderson CS Hackett ML. Frequency of post-stroke pneumonia: systematic review and meta-analysis of observational studies. Int J Stroke. (2018) 14:125–36. 10.1177/1747493018806196 30346258

[5] Westendorp WF Nederkoorn PJ Vermeij J-D Dijkgraaf MG van de Beek D. Post-stroke infection: a systematic review and meta-analysis. BMC Neurol. (2011) 11:110. 10.1186/1471-2377-11-110 21933425 PMC3185266

[6] Hong KS Kang DW Koo JS Yu KH Han MK Cho YJ. Impact of neurological and medical complications on 3-month outcomes in acute ischaemic stroke. Eur J Neurol. (2008) 15:1324–31. 10.1111/j.1468-1331.2008.02310.x 19049549

[7] Vermeij FH Scholte op Reimer WJ de Man P van Oostenbrugge RJ Franke CL de Jong G. Stroke-associated infection is an independent risk factor for poor outcome after acute ischemic stroke: data from the Netherlands stroke survey. Cerebrovasc Dis. (2009) 27:465–71. 10.1159/000210093 19329851

[8] Teh WH Smith CJ Barlas RS Wood AD Bettencourt-Silva JH Clark AB. Impact of stroke-associated pneumonia on mortality, length of hospitalization, and functional outcome. Acta Neurol Scand. (2018) 138:293–300. 10.1111/ane.12956 29749062

[9] Katzan IL Dawson NV Thomas CL Votruba ME Cebul RD. The cost of pneumonia after acute stroke. Neurology. (2007) 68:1938–43. 10.1212/01.wnl.0000263187.08969.45 17536051

[10] de Jonge JC van de Beek D Lyden P Brady MC Bath PM van der Worp HB. Temporal profile of pneumonia after stroke. Stroke. (2022) 53:53–60. 10.1161/STROKEAHA.120.032787 34517764 PMC8700305

[11] Kishore AK Vail A Bray BD Chamorro A Napoli MD Kalra L. Clinical risk scores for predicting stroke-associated pneumonia: a systematic review. Eur Stroke J. (2016) 1:76–84. 10.1177/2396987316651759 31008268 PMC6301233

[12] Ni J Shou W Wu X Sun J. Prediction of stroke-associated pneumonia by the A2DS2, AIS-APS, and ISAN scores: a systematic review and meta-analysis. Expert Rev Resp Med. (2021) 15:1–12. 10.1080/17476348.2021.1923482 33945394

[13] Zapata-Arriaza E Moniche F Blanca P-G Bustamante A Escudero-Martínez I Uclés O. External validation of the ISAN, A2DS2, and AIS-APS scores for predicting stroke-associated pneumonia. J Stroke Cerebrovasc Dis. (2018) 27:673–6. 10.1016/j.jstrokecerebrovasdis.2017.09.059 29103860

[14] Beam AL Kohane IS. Big data and machine learning in health care. JAMA. (2018) 319:1317. 10.1001/jama.2017.18391 29532063

[15] Ge Y Wang Q Wang L Wu H Peng C Wang J. Predicting post-stroke pneumonia using deep neural network approaches. Int J Med Inform. (2019) 132:103986. 10.1016/j.ijmedinf.2019.103986 31629312

[16] Li X Wu M Sun C Zhao Z Wang F Zheng X. Using machine learning to predict stroke-associated pneumonia in Chinese acute ischaemic stroke patients. Eur J Neurol. (2020) 27:1656–63. 10.1111/ene.14295 32374076

[17] Ruiz VM Goldsmith MP Shi L Simpao AF Gálvez JA Naim MY. Early prediction of clinical deterioration using data-driven machine-learning modeling of electronic health records. J Thorac Cardiovasc Surg. (2022) 164:211–22.e3. 10.1016/j.jtcvs.2021.10.060 34949457

[18] Sung S-F Hsieh C-Y Hu Y-H. Early prediction of functional outcomes after acute ischemic stroke using unstructured clinical text: retrospective cohort study. JMIR Med Inform. (2022) 10:e29806. 10.2196/29806 35175201 PMC8895286

[19] Tsui FR Shi L Ruiz V Ryan ND Biernesser C Iyengar S. Natural language processing and machine learning of electronic health records for prediction of first-time suicide attempts. JAMIA Open. (2021) 4:ooab011. 10.1093/jamiaopen/ooab011 33758800 PMC7966858

[20] Weissman GE Hubbard RA Ungar LH Harhay MO Greene CS Himes BE. Inclusion of unstructured clinical text improves early prediction of death or prolonged ICU stay. Crit Care Med. (2018) 46:1125–32. 10.1097/CCM.0000000000003148 29629986 PMC6005735

[21] Sung S Chen C Pan R Hu Y Jeng J. Natural language processing enhances prediction of functional outcome after acute ischemic stroke. J Am Heart Assoc. (2021) 10:e023486. 10.1161/JAHA.121.023486 34796719 PMC9075227

[22] Hsieh F-I Lien L-M Chen S-T Bai C-H Sun M-C Tseng H-P. Get with the guidelines-stroke performance indicators: surveillance of stroke care in the taiwan stroke registry. Circulation. (2010) 122:1116–23. 10.1161/CIRCULATIONAHA.110.936526 20805428

[23] Smith CJ Kishore AK Vail A Chamorro A Garau J Hopkins SJ. Diagnosis of stroke-associated pneumonia. Stroke. (2015) 46:2335–40. 10.1161/STROKEAHA.115.009617 26111886

[24] Bojanowski P Grave E Joulin A Mikolov T. Enriching word vectors with subword information. Transact Assoc Comput Linguis. (2017) 5:135–46. 10.1162/tacl_a_00051

[25] Devlin J Chang M-W Lee K Toutanova K. BERT: pre-training of deep bidirectional transformers for language understanding. NAACL HLT 2019 - 2019 Conference of the North American Chapter of the Association for Computational Linguistics: Human Language Technologies - Proceedings of the Conference. Minneapolis USA: Curran Associates, Inc. (2019). p. 4171–86

[26] Schwartz HA Eichstaedt JC Kern ML Dziurzynski L Ramones SM Agrawal M. Personality, gender, and age in the language of social media: the open-vocabulary approach. PLoS ONE. (2013) 8:e73791. 10.1371/journal.pone.0073791 24086296 PMC3783449

[27] Mujtaba G Shuib L Idris N Hoo WL Raj RG Khowaja K. Clinical text classification research trends: systematic literature review and open issues. Expert Syst Appl. (2019) 116:494–520. 10.1016/j.eswa.2018.09.034

[28] Deng X Li Y Weng J Zhang J. Feature selection for text classification: a review. Multimed Tools Appl. (2018) 78:3797–816. 10.1007/s11042-018-6083-534404964

[29] Culpeper J. Keyness: words, parts-of-speech and semantic categories in the character-talk of Shakespeare's Romeo and Juliet. Int J Corpus Linguis. (2009) 14:29–59. 10.1075/ijcl.14.1.03cul

[30] Zhang Y Chen Q Yang Z Lin H Lu Z. BioWordVec, improving biomedical word embeddings with subword information and MeSH. Sci Data. (2019) 6:52. 10.1038/s41597-019-0055-0 31076572 PMC6510737

[31] Chen Q Peng Y Lu Z. BioSentVec: creating sentence embeddings for biomedical texts. 2019 IEEE Int Conf Healthc Informatics ICHI. (2019) 00:1–5. 10.1109/ICHI.2019.8904728

[32] Alsentzer E Murphy J Boag W Weng W-H Jindi D Naumann T. Publicly available clinical BERT embeddings. Proc 2nd Clin Nat Lang Process Work. (2019) pp. 72–78. 10.18653/v1/W19-1909

[33] Hoffmann S Malzahn U Harms H Koennecke H-C Berger K Kalic M. Development of a clinical score (A2DS2) to predict pneumonia in acute ischemic stroke. Stroke. (2012) 43:2617–23. 10.1161/STROKEAHA.112.653055 22798325

[34] Smith CJ Bray BD Hoffman A Meisel A Heuschmann PU Wolfe CDA. Can a novel clinical risk score improve pneumonia prediction in acute stroke care? A UK multicenter cohort study. J Am Heart Assoc. (2015) 4:e001307. 10.1161/JAHA.114.001307 25587017 PMC4330058

[35] Friedant AJ Gouse BM Boehme AK Siegler JE Albright KC Monlezun DJ. A simple prediction score for developing a hospital-acquired infection after acute ischemic stroke. J Stroke Cerebrovasc Dis. (2015) 24:680–6. 10.1016/j.jstrokecerebrovasdis.2014.11.014 25601173 PMC4359649

[36] Kumar S Marchina S Massaro J Feng W Lahoti S Selim M. ACDD4 score: a simple tool for assessing risk of pneumonia after stroke. J Neurol Sci. (2017) 372:399–402. 10.1016/j.jns.2016.10.050 27823836

[37] Branco P Torgo L Ribeiro RP A. survey of predictive modeling on imbalanced domains. ACM Comput Surv (CSUR). (2016) 49:1–50. 10.1145/2907070

[38] Lundberg SM Erion G Chen H DeGrave A Prutkin JM Nair B. From local explanations to global understanding with explainable AI for trees. Nat Mach Intell. (2020) 2:56–67. 10.1038/s42256-019-0138-9 32607472 PMC7326367

[39] Haixiang G Yijing L Shang J Mingyun G Yuanyue H Bing G. Learning from class-imbalanced data: review of methods and applications. Expert Syst Appl. (2017) 73:220–39. 10.1016/j.eswa.2016.12.035

[40] DeLong ER DeLong DM Clarke-Pearson DL. Comparing the areas under two or more correlated receiver operating characteristic curves: a nonparametric approach. Biometrics. (1988) 44:837–45. 10.2307/2531595 3203132

[41] LaValley MP. Logistic regression. Circulation. (2008) 117:2395–9. 10.1161/CIRCULATIONAHA.106.682658 18458181

[42] Habibzadeh F Habibzadeh P Yadollahie M. On determining the most appropriate test cut-off value: the case of tests with continuous results. Biochem Medica. (2016) 26:297–307. 10.11613/BM.2016.034 27812299 PMC5082211

[43] Steyerberg EW Vickers AJ Cook NR Gerds T Gonen M Obuchowski N. Assessing the performance of prediction models: a framework for traditional and novel measures. Epidemiology. (2010) 21:128–38. 10.1097/EDE.0b013e3181c30fb2 20010215 PMC3575184

[44] Li W He C. Association of platelet-to-lymphocyte ratio with stroke-associated pneumonia in acute ischemic stroke. J Healthc Eng. (2022) 2022:1033332. 10.1155/2022/1033332 35340256 PMC8956427

[45] Quesada AS Aliaga AÁ Julia Saumell JB Galano MEH. Relationship between indicators of nutritional status and the development of pneumonia associated with ischemic stroke. Finlay. (2020) 10:231–9.

[46] Ogliari G Mahinrad S Stott DJ Jukema JW Mooijaart SP Macfarlane PW. Resting heart rate, heart rate variability and functional decline in old age. CMAJ. (2015) 187:E442–9. 10.1503/cmaj.150462 26323697 PMC4610849

[47] Kuo Y-W Lee M Huang Y-C Lee J-D. Initial in-hospital heart rate is associated with three-month functional outcomes after acute ischemic stroke. BMC Neurol. (2021) 21:222. 10.1186/s12883-021-02252-2 34116663 PMC8194208

[48] Staaf G Samuelsson M Lindgren A Norrving B. Sensorimotor stroke; clinical features, MRI findings, and cardiac and vascular concomitants in 32 patients. Acta Neurol Scand. (1998) 97:93–8. 10.1111/j.1600-0404.1998.tb00616.x 9517858

[49] Arboix A García-Plata C García-Eroles L Massons J Comes E Oliveres M. Clinical study of 99 patients with pure sensory stroke. J Neurol. (2005) 252:156–62. 10.1007/s00415-005-0622-5 15729520

[50] Derex L Adeleine P Nighoghossian N Honnorat J Trouillas P. Factors influencing early admission in a french stroke unit. Stroke. (2002) 33:153–9. 10.1161/hs0102.100533 11779905

[51] Stanley D Mason LJ Mackin KE Srikhanta YN Lyras D Prakash MD. Translocation and dissemination of commensal bacteria in post-stroke infection. Nat Med. (2016) 22:1277–84. 10.1038/nm.4194 27694934

[52] Shi K Wood K Shi F-D Wang X Liu Q. Stroke-induced immunosuppression and poststroke infection. Stroke Vasc Neurol. (2018) 3:34–41. 10.1136/svn-2017-000123 29600006 PMC5870641

[53] Smith EE Kent DM Bulsara KR Leung LY Lichtman JH Reeves MJ. Effect of dysphagia screening strategies on clinical outcomes after stroke. Stroke. (2018) 49:e123–8. 10.1161/STR.0000000000000159 29367332

[54] Vermeij J Westendorp WF Dippel DW van de Beek D Nederkoorn PJ. Antibiotic therapy for preventing infections in people with acute stroke. Cochrane Database Syst Rev. (2018) 2018:CD008530. 10.1002/14651858.CD008530.pub3 29355906 PMC6491314

[55] Westendorp WF Vermeij J-D Smith CJ Kishore AK Hodsoll J Kalra L. Preventive antibiotic therapy in acute stroke patients: a systematic review and meta-analysis of individual patient data of randomized controlled trials. Eur Stroke J. (2021) 6:385–94. 10.1177/23969873211056445 35342808 PMC8948510

[56] Lyons M Smith C Boaden E Brady MC Brocklehurst P Dickinson H. Oral care after stroke: where are we now? Eur Stroke J. (2018) 3:347–54. 10.1177/2396987318775206 31236482 PMC6571511

[57] Yuan D Zhang J Wang X Chen S Wang Y. Intensified oral hygiene care in stroke-associated pneumonia: a pilot single-blind randomized controlled trial. Inquiry. (2020) 57:0046958020968777. 10.1177/0046958020968777 33124506 PMC7607750

[58] Li J Liu L Le TD Liu J. Accurate data-driven prediction does not mean high reproducibility. Nat Mach Intell. (2020) 2:13–5. 10.1038/s42256-019-0140-2

