# Supplementary material for: Application of machine learning and natural language processing for predicting stroke-associated pneumonia
Source: Front Public Health. 2022 Sep 29;10:1009164. doi: 10.3389/fpubh.2022.1009164 (PMC9556866; doi:10.3389/fpubh.2022.1009164)
Supplement: Supplementary file 1 [file Data_Sheet_1.PDF]

Supplementary Material

Supplementary Table 1. General specifics of the data sources used in this study (up to Apr 2022).

	Hospital stroke registry	Ditmanson Research Database
Time period	Oct 2007 to Apr 2022	Nov 2005 to Apr 2022
Patient number	Over 12,000	Over 1.4 million
Inclusion criteria	Patients admitted to the study hospital for any type of stroke within 10 days of stroke onset	Patients who made visits to the study hospital
Categories of data	Structured data<br>• Demographics<br>• Risk factors<br>• Symptoms and signs<br>• Clinical assessment<br>• Stroke type<br>• Laboratory results<br>• Imaging findings<br>• Treatments and interventions<br>• Complications<br>• Outcomes	Structured data<br>• Demographics<br>• Vital signs<br>• Diagnoses<br>• Prescriptions<br>• Procedures<br>• Laboratory results<br>Textual data<br>• Physician notes<br>• Nursing notes<br>• Laboratory reports<br>• Radiology reports<br>• Pathology reports

Supplementary Table 2. Structured variables used for the construction of machine learning models in the study population (N = 5913).

Variable	Value	Missing, n (%)
Demographics		
Age	70 (59–78)	0 (0%)
Male	3643 (61.6)	0 (0%)
Pre-stroke functional status		
Pre-stroke dependency	562 (9.5)	0 (0%)
Pre-stroke mRS	0 (0–0)	0 (0%)
Risk factors and comorbidities		
Hypertension	4739 (80.2)	0 (0%)
Diabetes	2422 (41.0)	0 (0%)
Hyperlipidemia	3167 (53.6)	0 (0%)
Atrial fibrillation	822 (13.9)	0 (0%)
Congestive heart failure	226 (3.8)	0 (0%)
COPD	397 (6.7)	0 (0%)
Smoking	2431 (41.1)	0 (0%)
Ischemic heart disease	557 (9.4)	0 (0%)
Recent myocardial infarction	347 (5.9)	0 (0%)
Prior stroke or TIA	1267 (21.4)	0 (0%)
ESRD	173 (2.9)	0 (0%)
Cancer	431 (7.3)	0 (0%)
Peripheral artery disease	180 (3.0)	0 (0%)
Valvular heart disease	53 (0.9)	0 (0%)
Prior use of medications		
Antiplatelet agents	1099 (18.6)	0 (0%)
Oral anticoagulants	139 (2.4)	0 (0%)
Antihypertensive agents	3073 (52.0)	0 (0%)
Physiological measurements		
Height, m	160 (153–166)	3 (0.05%)
Weight, kg	62.6 (54.0–71.3)	4 (0.07%)
Body mass index (kg/m^2)	24.53 (21.92–27.26)	4 (0.07%)
Systolic BP (mmHg)	163 (146–189)	0 (0%)
Diastolic BP (mmHg)	94 (83–107)	1 (0.02%)
Body temperature (℃)	36.4 (36.0–36.8)	3 (0.05%)
Heart rate (beats per minute)	80 (70–93)	4 (0.07%)
Respiratory rate (breaths per minute)	20 (18–20)	7 (0.12%)
Neurological assessment		
NIHSS	5 (3–11)	0 (0%)

GCS - total score	15 (14–15)	0 (0%)
GCS - eye component score	4 (4–4)	0 (0%)
GCS - motor component score	6 (6–6)	0 (0%)
GCS - verbal component score	5 (5–5)	0 (0%)
Dysphagia	1195 (20.2)	0 (0%)
Dysarthria	3039 (51.4)	0 (0%)
Blood tests		
Glucose, mmol/L	7.38 (6.11–9.99)	64 (1.08%)
Glycosylated hemoglobin	0.063 (0.058–0.079)	2037 (34.45%)
Urea nitrogen, mmol/L	0.61 (0.49–0.79)	135 (2.28%)
Creatinine, μmol/L	83.10 (64.53–106.08)	64 (1.08%)
AST, μkat/L	0.38 (0.32–0.52)	1302 (22.02%)
ALT, μkat/L	0.35 (0.25–0.50)	241 (4.08%)
Total cholesterol, mmol/L	4.60 (3.93–5.30)	614 (10.38%)
High-density lipoprotein, mmol/L	1.14 (0.93–1.37)	991 (16.76%)
Low-density lipoprotein, mmol/L	2.84 (2.28–3.49)	874 (14.78%)
Triglyceride, mmol/L	1.22 (0.86–1.75)	595 (10.06%)
White blood cells, 10^9/L	7.68 (6.19–9.61)	0 (0%)
Hemoglobin, mmol/L	2.15 (1.92–2.34)	2 (0.03%)
Hematocrit	0.41 (0.37–0.44)	3 (0.05%)
Platelet count, 10^9/L	204 (168–245)	5 (0.08%)
INR	1.02 (0.98–1.07)	437 (7.39%)
APTT, s	28.4 (25.7–32.3)	461 (7.80%)
Other		
Intravenous thrombolysis	455 (7.7)	0 (0%)
Onset-to-door time ≥ 3 h	4385 (74.2)	0 (0%)

Data are given as n (%) and median (interquartile range).

ALT, alanine aminotransferase; APTT, activated partial thromboplastin time; AST, aspartate aminotransferase; BP, blood pressure; COPD, chronic obstructive pulmonary disease; ESRD, end-stage renal disease; GCS, Glasgow coma scale; INR, international normalization ratio; mRS, modified Rankin scale; NIHSS, National Institutes of Health Stroke Scale; TIA, transient ischemic attack.

Supplementary Table 3. Baseline characteristics of the training and holdout test sets.

Characteristic	Total (N = 5913)	Training (N = 4434)	Holdout test (N = 1479)	*P*
Age	70 (59–78)	70 (59–78)	70 (59–78)	0.781
Male	3643 (61.6)	2739 (61.8)	904 (61.1)	0.656
Hypertension	4739 (80.2)	3557 (80.2)	1182 (79.9)	0.801
Diabetes	2422 (41.0)	1855 (41.8)	567 (38.3)	0.018
Hyperlipidemia	3167 (53.6)	2394 (54.0)	773 (52.3)	0.249
AF	822 (13.9)	626 (14.1)	196 (13.3)	0.405
CHF	226 (3.8)	170 (3.8)	56 (3.8)	0.934
COPD	397 (6.7)	299 (6.7)	98 (6.6)	0.876
Smoking	2431 (41.1)	1815 (40.9)	616 (41.7)	0.628
Pre-stroke dependency	562 (9.5)	428 (9.7)	134 (9.1)	0.501
Pre-stroke mRS	0 (0–0)	0 (0–0)	0 (0–0)	0.331
NIHSS	5 (3–11)	6 (3–11)	5 (3–10)	0.022
GCS	15 (14–15)	15 (14–15)	15 (15–15)	0.026
Dysphagia	1195 (20.2)	914 (20.6)	281 (19.0)	0.181
Dysarthria	3039 (51.4)	2315 (52.2)	724 (49.0)	0.030
Glucose (mmol/L)	7.38 (6.11–9.99)	7.41 (6.11–9.99)	7.27 (6.05–10.10)	0.659
WBC (10^9/L)	7.68 (6.19–9.61)	7.70 (6.21–9.64)	7.63 (6.13–9.52)	0.438
A^2DS^2	4 (1–5)	4 (1–5)	3 (1–5)	0.023
ISAN	7 (4–10)	7 (4–10)	7 (4–10)	0.093
PNA	4 (1–5)	4 (1–5)	4 (1–5)	0.029
$ACDD^4$	1 (0–2)	1 (0–2)	1 (0–2)	0.042
SAP	450 (7.6)	338 (7.6)	112 (7.6)	0.950

Data are given as n (%) and median (interquartile range).
AF, atrial fibrillation; CHF, congestive heart failure; COPD, chronic obstructive pulmonary disease; GCS, Glasgow coma scale; mRS, modified Rankin Scale; NIHSS, National Institutes of Health Stroke Scale; SAP, stroke-associated pneumonia; WBC, white blood cell.

Supplementary Table 4. Performance of random forest ML models for predicting SAP on the holdout test set.

Model	Data	Vectorization	Resampling	No of trees	AUC	Accuracy	Precision	Recall	F1 score
Structured[†]	Structured	-	1:1 under-sampling	900	0.828	76.3%	0.212	0.786	0.334
Text-BOW-TF	Text	TF	1:2 under-sampling	1400	0.717	76.3%	0.164	0.518	0.249
Text-BOW-BR	Text	BR	1:1 under-sampling	400	0.686	66.5%	0.137	0.643	0.225
Text-BOW-TFIDF	Text	TF-IDF	1:1 under-sampling	1100	0.728	67.8%	0.153	0.714	0.252
Text-BOW-Bigram	Text	Bigram	1:1 under-sampling	1500	0.731	66.3%	0.148	0.723	0.245
Text-fastText	Text	fastText	1:1 oversampling	200	0.711	81.5%	0.154	0.321	0.208
Text-BERT	Text	BERT	1:1 under-sampling	1200	0.720	66.9%	0.143	0.679	0.237
Both-BOW-TF	Both	TF	1:3 under-sampling	1300	0.835	89.0%	0.324	0.411	0.362
Both-BOW-BR[‡]	Both	BR	1:2 under-sampling	1900	0.840	83.2%	0.254	0.634	0.363
Both-BOW-TFIDF	Both	TF-IDF	1:1 under-sampling	1000	0.826	76.8%	0.211	0.750	0.329
Both-BOW-Bigram	Both	Bigram	1:2 under-sampling	2000	0.839	83.6%	0.264	0.652	0.375
Both-fastText	Both	fastText	1:1 under-sampling	2000	0.800	73.2%	0.183	0.732	0.292
Both-BERT	Both	BERT	1:1 oversampling	200	0.782	92.4%	0.500	0.018	0.034

[†]ML Model B; [‡]ML Model A.
AUC, area under the receiver operating characteristic curve; BERT, bidirectional encoder representations from transformers; BOW, bag-of-words; BR, binary representation; ML, machine learning; SAP, stroke-associated pneumonia; TF, term frequency; TF-IDF, term frequency with inverse document frequency.

Supplementary Table 5. *P* values for pairwise comparisons of AUCs between ML models.

Model	Text-BOW-TF	Text-BOW-BR	Text-BOW-TFIDF	Text-BOW-Bigram	Text-fastText	Text-BERT	Both-BOW-TF	Both-BOW-BR[‡]	Both-BOW-TFIDF	Both-BOW-Bigram	Both-fastText	Both-BERT
Structured[†]	<0.001	<0.001	<0.001	<0.001	<0.001	<0.001	0.270	0.040	0.809	0.056	0.017	0.002
Text-BOW-TF	-	0.031	0.478	0.233	0.739	0.881	<0.001	<0.001	<0.001	<0.001	<0.001	0.003
Text-BOW-BR	-	-	0.013	0.007	0.215	0.078	<0.001	<0.001	<0.001	<0.001	<0.001	<0.001
Text-BOW-TFIDF	-	-	-	0.843	0.313	0.641	<0.001	<0.001	<0.001	<0.001	<0.001	0.004
Text-BOW-Bigram	-	-	-	-	0.297	0.615	<0.001	<0.001	<0.001	<0.001	<0.001	0.019
Text-fastText	-	-	-	-	-	0.484	<0.001	<0.001	<0.001	<0.001	<0.001	<0.001
Text-BERT	-	-	-	-	-	-	<0.001	<0.001	<0.001	<0.001	<0.001	<0.001
Both-BOW-TF	-	-	-	-	-	-	-	0.203	0.218	0.192	0.001	<0.001
Both-BOW-BR[‡]	-	-	-	-	-	-	-	-	0.030	0.767	<0.001	<0.001
Both-BOW-TFIDF	-	-	-	-	-	-	-	-	-	0.029	0.005	0.002
Both-BOW-Bigram	-	-	-	-	-	-	-	-	-	-	<0.001	<0.001
Both-fastText	-	-	-	-	-	-	-	-	-	-	-	0.128

[†]ML Model B; [‡]ML Model A.

AUC, area under the receiver operating characteristic curve; BERT, bidirectional encoder representations from transformers; BOW, bag-of-words; BR, binary representation; ML, machine learning; TF, term frequency; TF-IDF, term frequency with inverse document frequency.

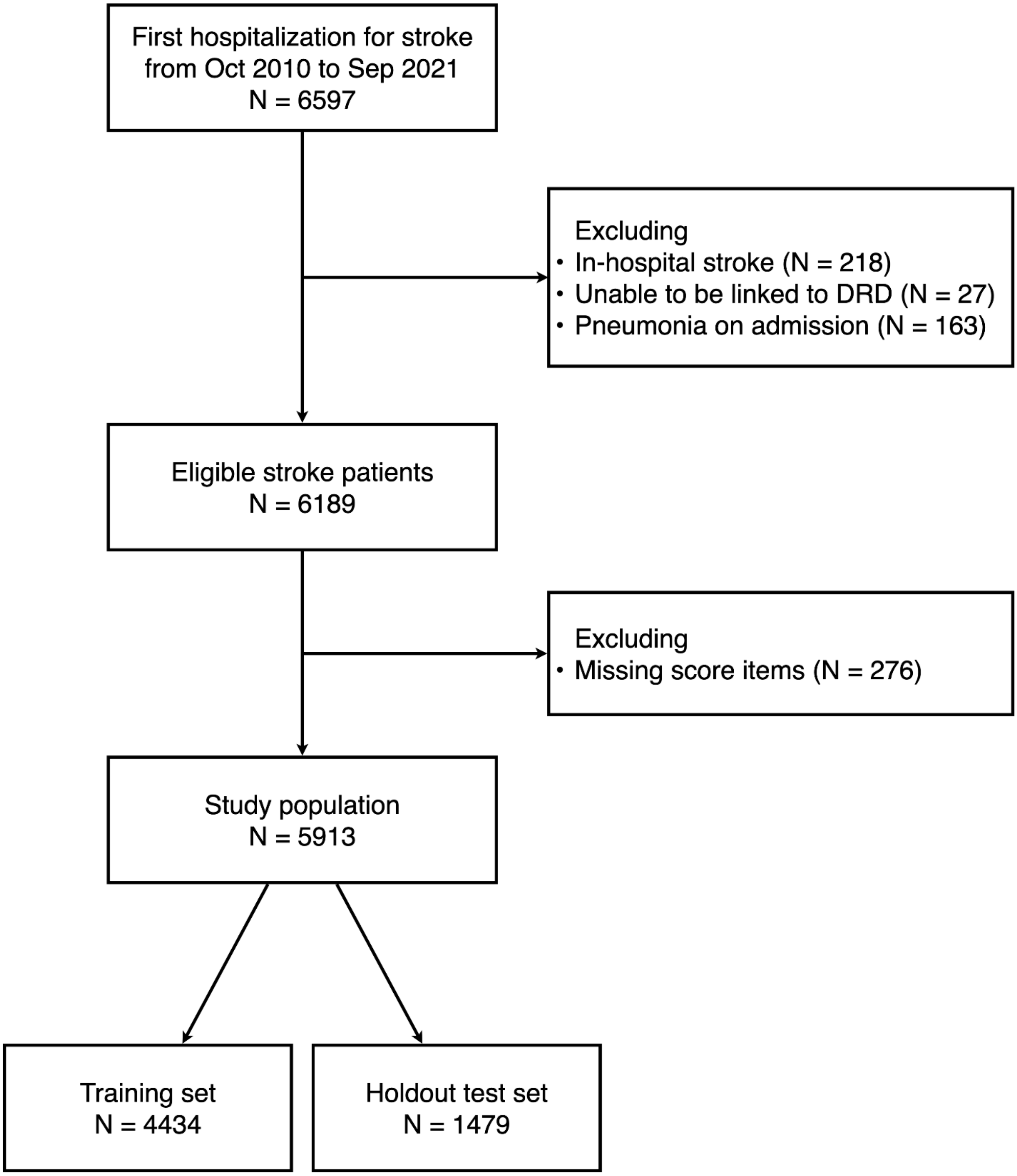

Supplementary Figure 1. Flowchart showing the derivation of the study population. DRD, Ditmanson Research Database.

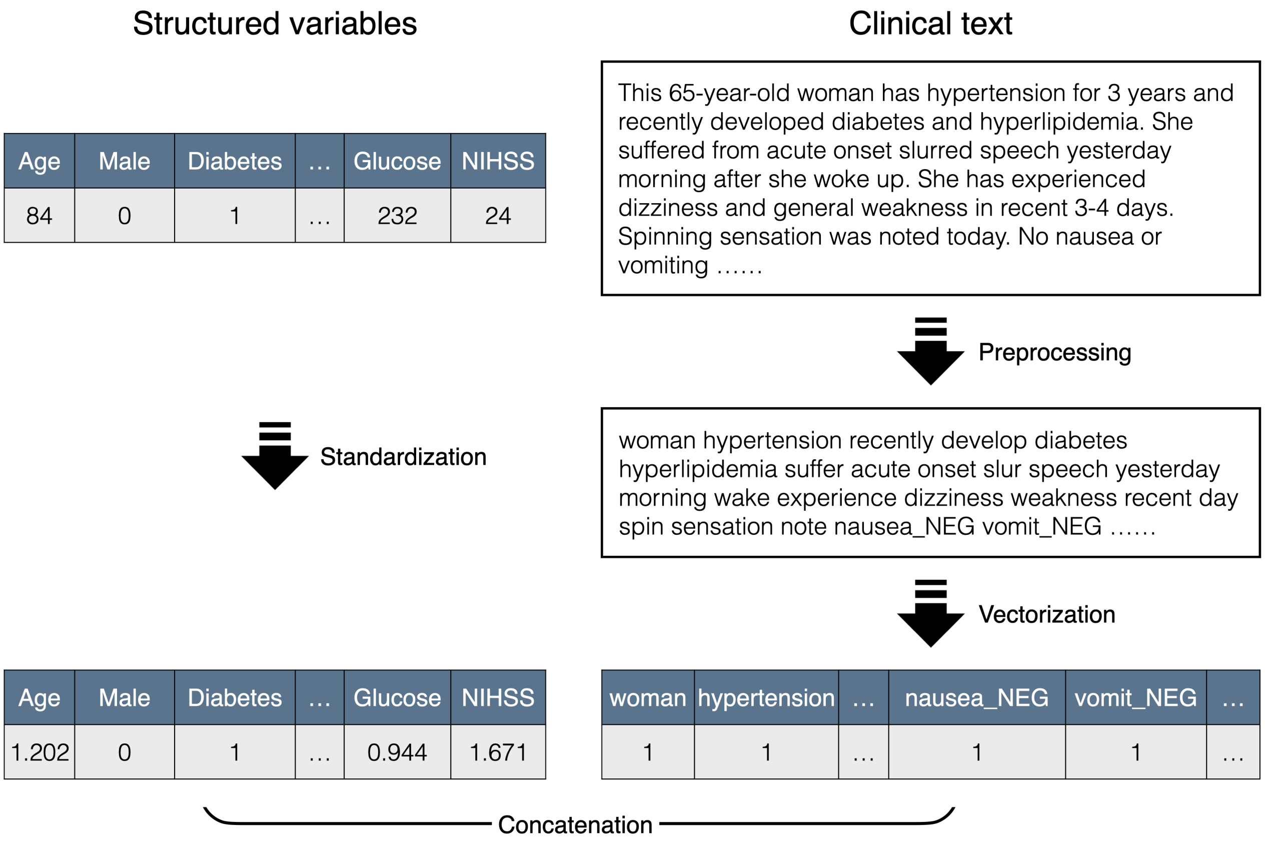

Supplementary Figure 2. An example of feature extraction and preprocessing using the bag-of-words approach.

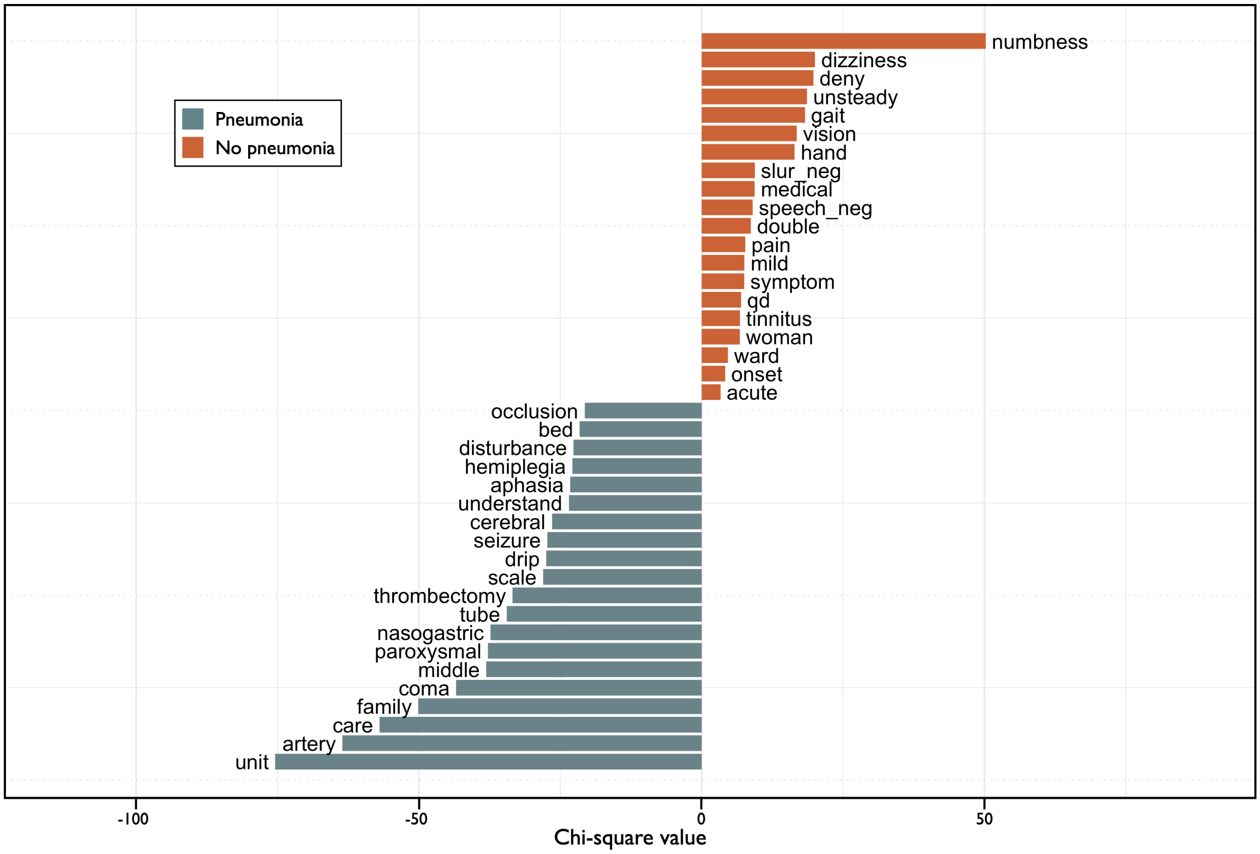

Supplementary Figure 3. Keyness plots showing the top 20 words that frequently appear in the documents of patients with stroke-associated pneumonia and those without based on chi-square statistics for bag-of-words model (term frequency). A negated word is suffixed with "_neg."

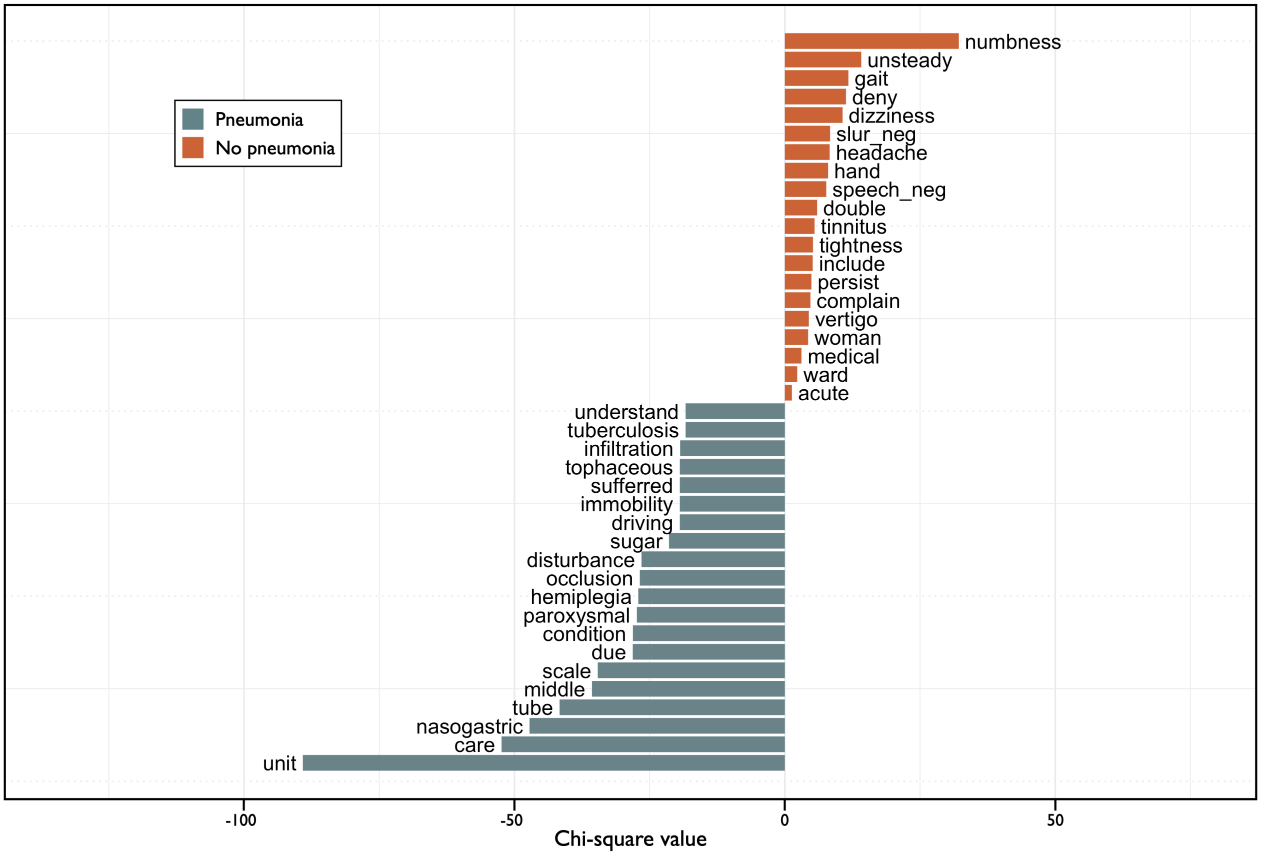

Supplementary Figure 4. Keyness plots showing the top 20 words that frequently appear in the documents of patients with stroke-associated pneumonia and those without based on chi-square statistics for bag-of-words model (binary representation). A negated word is suffixed with "_neg."

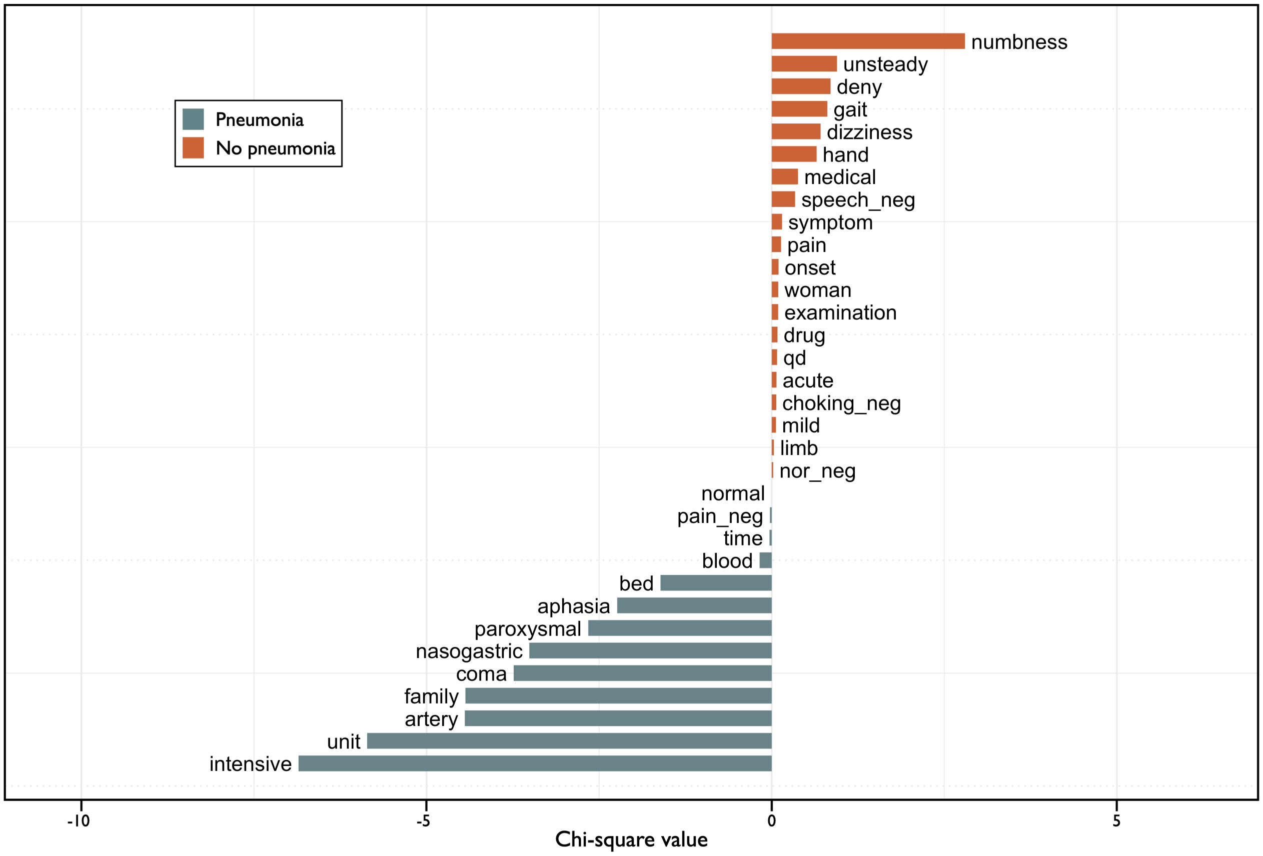

Supplementary Figure 5. Keyness plots showing the top 20 words that frequently appear in the documents of patients with stroke-associated pneumonia and those without based on chi-square statistics for bag-of-words model (term frequency with inverse document frequency weighting). A negated word is suffixed with "_neg."

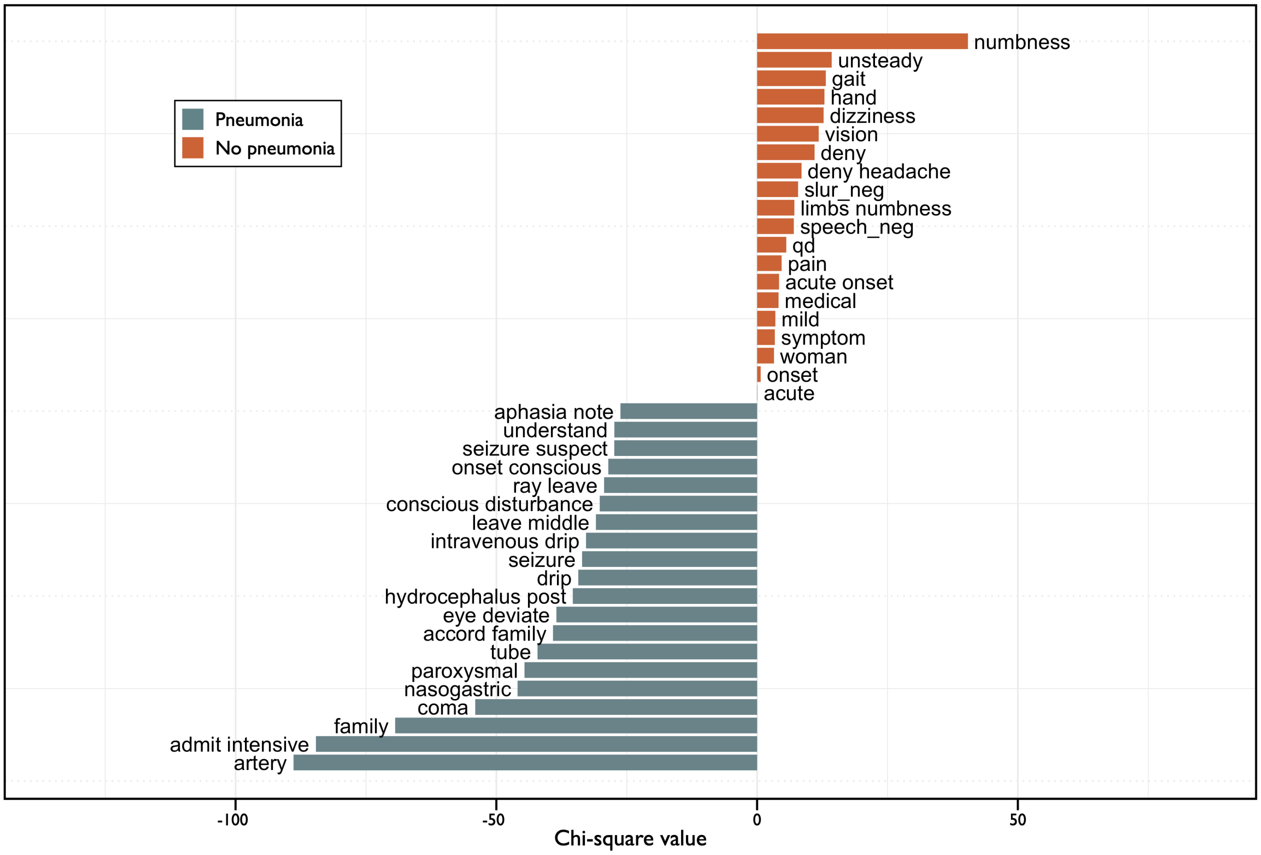

Supplementary Figure 6. Keyness plots showing the top 20 words or phrases that frequently appear in the documents of patients with stroke-associated pneumonia and those without based on chi-square statistics for bag-of-words model (bigram). A negated word or phrase is suffixed with "_neg."

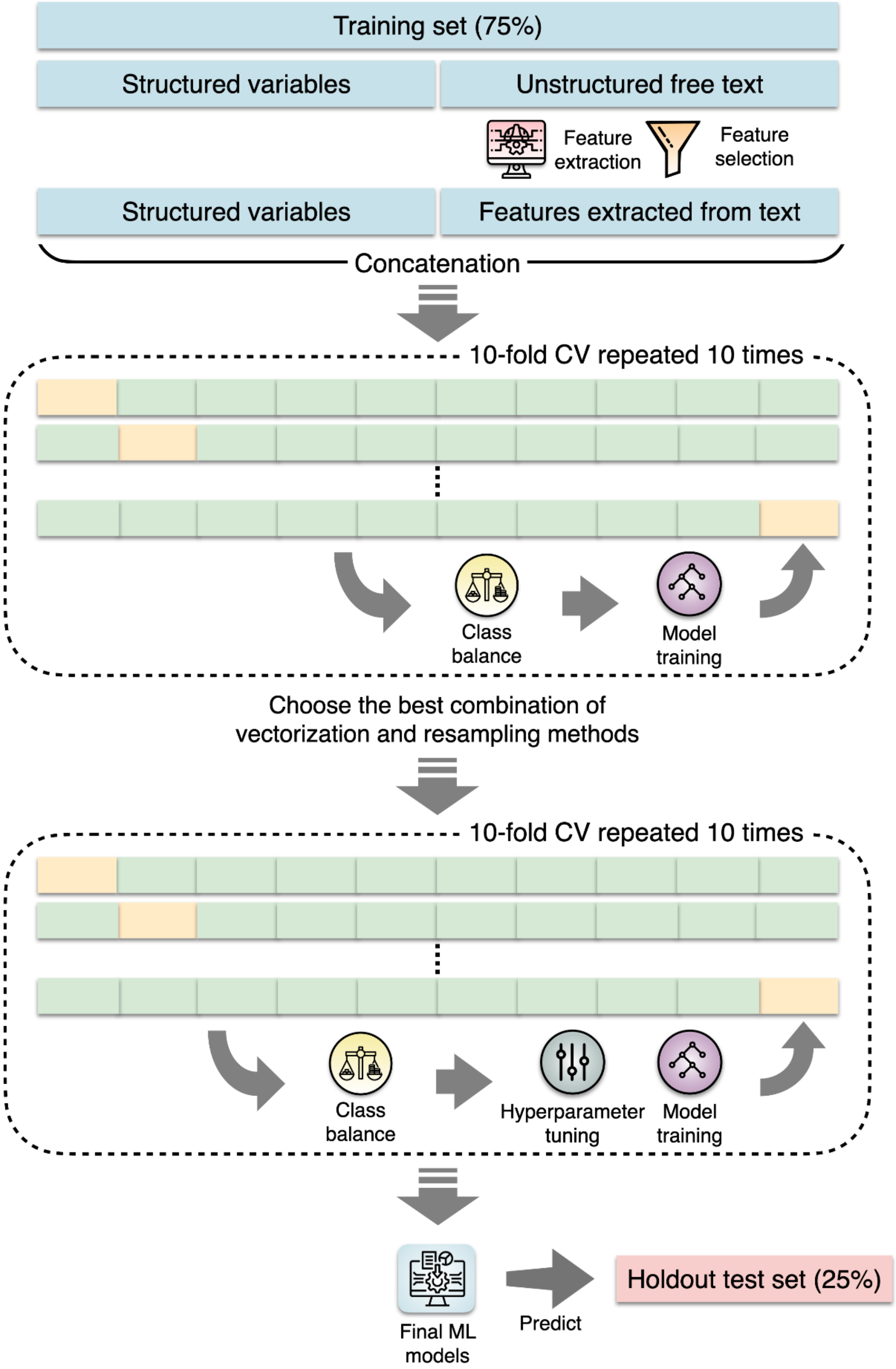

Supplementary Figure 7. The process of machine learning model construction. During the training process, we repeated 10-fold CV 10 times to obtain a reliable estimate of the AUC. The first 10-fold CV in the middle of the figure was used to determine the best combination of text vectorization and resampling methods. The second 10-fold CV in the bottom of the figure was used to determine the best number of decision trees in the random forest. AUC, area under the receiver operating characteristic curve; CV, cross validation; ML, machine learning.

A	Structured	Features from Text						Combined					
		TF	BR	TF-IDF	Bigram	fastText	BERT	TF	BR	TF-IDF	Bigram	fastText	BERT
Original data	0.7683	0.6840	0.6972	0.6930	0.6790	0.6635	0.6692	0.7803	0.7842	0.7705	0.7814	0.7482	0.7453
Oversampled (1:1)	0.7761	0.6897	0.6943	0.7081	0.6748	0.7113	0.7189	0.7886	0.7896	0.7887	0.7903	0.7697	0.7797
Oversampled (1:2)	0.7803	0.6871	0.6957	0.7021	0.6728	0.7028	0.7142	0.7915	0.7957	0.7928	0.7921	0.7751	0.7763
Oversampled (1:3)	0.7803	0.6888	0.6969	0.6982	0.6746	0.6944	0.7082	0.7916	0.7947	0.7876	0.7943	0.7677	0.7695
Undersampled (1:1)	0.7870	0.7050	0.7153	0.7193	0.6996	0.7094	0.7201	0.7966	0.7972	0.7948	0.7969	0.7754	0.7706
Undersampled (1:2)	0.7833	0.7053	0.7152	0.7149	0.6960	0.7025	0.7111	0.7967	0.7973	0.7916	0.7970	0.7712	0.7706
Undersampled (1:3)	0.7816	0.7014	0.7106	0.7112	0.6934	0.6936	0.7056	0.7971	0.7951	0.7899	0.7952	0.7687	0.7686

B	Structured	Features from Text						Combined					
		TF	BR	TF-IDF	Bigram	fastText	BERT	TF	BR	TF-IDF	Bigram	fastText	BERT
Original data	0.7010	0.7375	0.7579	0.7591	0.7474	0.7324	0.7087	0.7284	0.7434	0.7140	0.7352	0.7177	0.7287
Oversampled (1:1)	0.7682	0.7435	0.7570	0.7617	0.7525	0.7307	0.7225	0.7708	0.7831	0.7808	0.7782	0.7717	0.7654
Oversampled (1:2)	0.7488	0.7388	0.7571	0.7618	0.7478	0.7299	0.7156	0.7587	0.7735	0.7655	0.7642	0.7495	0.7493
Oversampled (1:3)	0.7460	0.7357	0.7559	0.7612	0.7473	0.7253	0.7097	0.7588	0.7714	0.7637	0.7624	0.7472	0.7428
Undersampled (1:1)	0.7041	0.7081	0.7262	0.7538	0.7125	0.7005	0.7000	0.7201	0.7269	0.7183	0.7221	0.7266	0.7403
Undersampled (1:2)	0.6953	0.7104	0.7408	0.7582	0.7257	0.7117	0.6739	0.7109	0.7199	0.7063	0.7249	0.7086	0.7362
Undersampled (1:3)	0.6928	0.719	0.7450	0.7598	0.7330	0.7218	0.6824	0.7123	0.7304	0.7014	0.7281	0.7182	0.7362

Supplementary Figure 8. Heat maps showing AUC values across machine learning models using random forest classifier (A) or logistic regression classifier (B) with different combinations of text vectorization techniques and class balance methods. AUC, area under the receiver operating characteristic curve; BERT, bidirectional encoder representations from transformers; BR, binary representation; TF, term frequency; TF-IDF, term frequency with inverse document frequency.

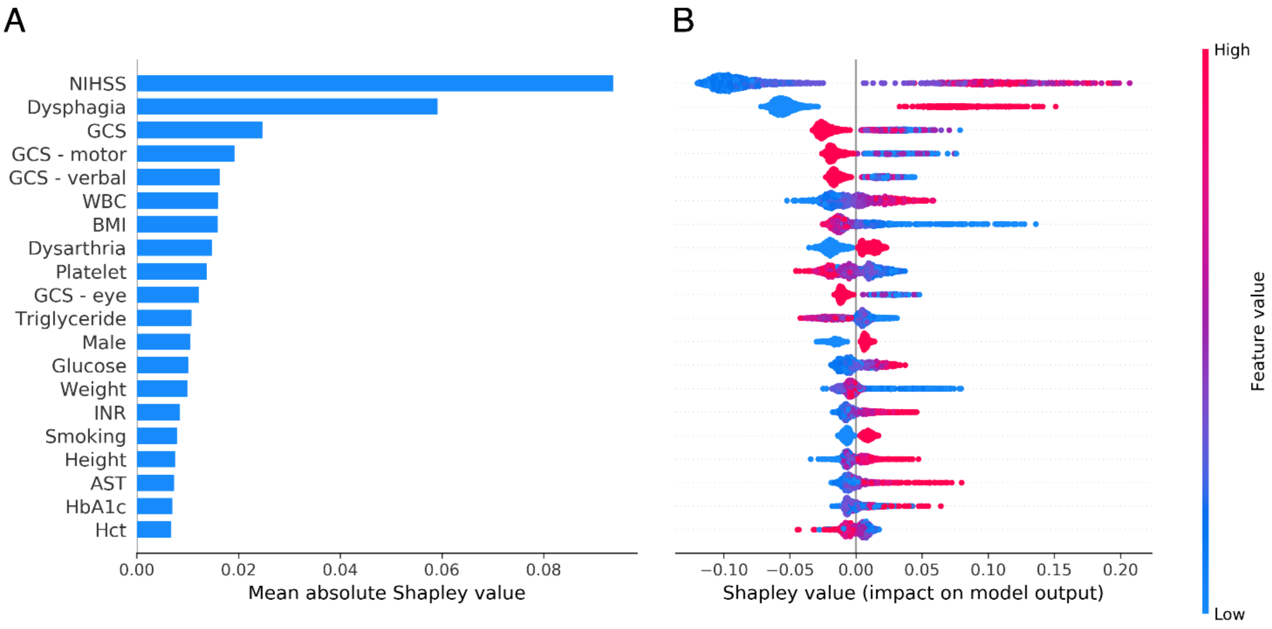

Supplementary Figure 9. The top 20 most influential features identified by the model based on structured variables alone. The average impact of each feature on the model output was quantified as mean absolute Shapley values (A). Each feature's individual Shapley values for each patient are depicted in a beeswarm plot (B), where a dot's position on the x-axis denotes each feature's contribution to the model prediction for the corresponding patient. The color of the dot specifies the relative value of the corresponding feature. AST, aspartate aminotransferase; BMI, body mass index; GCS, Glasgow coma scale; HbA1c, glycated hemoglobin; Hct, hematocrit; HR, heart rate; INR, international normalization ratio; NIHSS, National Institutes of Health Stroke Scale; WBC, white blood cells.

Supplementary Methods

A receiver operating characteristic (ROC) curve is a graph showing the performance of a classification model at all classification thresholds. This curve plots two parameters, which are true positive rate (TPR) and false positive rate (FPR). TPR (sensitivity) is defined as true positives (TPs) divided by the sum of TPs and false negatives (FNs). FPR (1 - specificity) is defined as false positives (FPs) divided by the sum of FPs and true negatives (TNs). Then the ROC curve plots TPR vs. FPR at different classification thresholds. Take one of the stroke-associated pneumonia (SAP) risk scores, PNA score, for example. The following table lists the distribution of the PNA score among our patients.

PNA Score	0	1	2	3	4	5	6	7
SAP	0	8	1	5	24	20	36	18
No SAP	160	259	74	185	371	167	114	37

The score thresholds are dictated by the data. Because the PNA score is an integer-based score ranging from 0 to 7, the thresholds are set at the middle of two consecutive score values. Therefore, the corresponding thresholds are 0.5, 1.5, 2.5, 3.5, 4.5, 5.5, and 6.5. The following table lists the TPR and FPR at all classification thresholds.

Score threshold	0.5	1.5	2.5	3.5	4.5	5.5	6.5
TPs	112	104	103	98	74	54	18
FPs	1207	948	874	689	318	151	37
TNs	160	419	493	678	1049	1216	1330
FNs	0	8	9	14	38	58	94
TPR	1	0.929	0.920	0.875	0.661	0.482	0.161
FPR	0.883	0.693	0.639	0.504	0.233	0.110	0.027

Without transformation of the PNA score, the ROC curve will be:

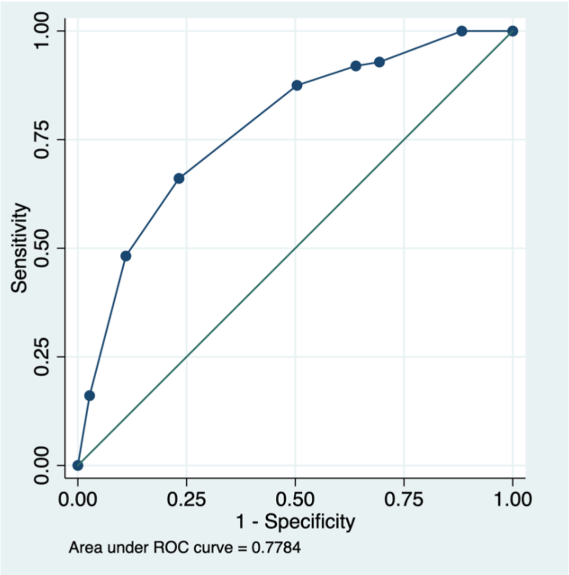

If we transform the PNA score into a binary variable using the cut-off value of 4.5 points, then the distribution is shown in the following table.

PNA score	<4.5	≥4.5
SAP	38	74
No SAP	1049	318

The following table lists the TPR and FPR.

Score threshold	4.5
TPs	74
FPs	318
TNs	1049
FNs	38
TPR	0.661
FPR	0.233

Then the ROC curve will be:

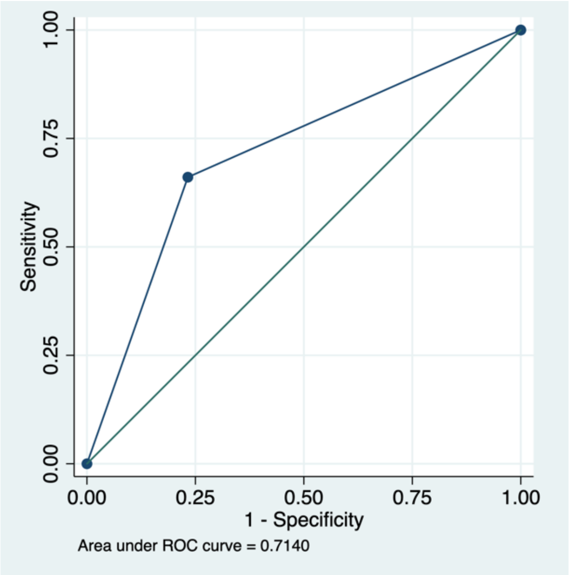

In our study, we performed the ROC analysis using the first approach. In other words, we did not perform any transformation on the SAP risk scores when calculating the AUC. However, in order to calculate accuracy, precision, recall, and F1 score, we performed transformation to binarize the SAP risk scores. The cut-off value for each SAP risk score was determined by finding the point on the ROC curve with minimum distance from the left-upper corner. For example, in the following figure showing the ROC of the PNA score, the point at the cut-off value of 4.5 has the minimum distance from the left-upper corner. Therefore, 4.5 was determined to be the cut-off value for the PNA score.

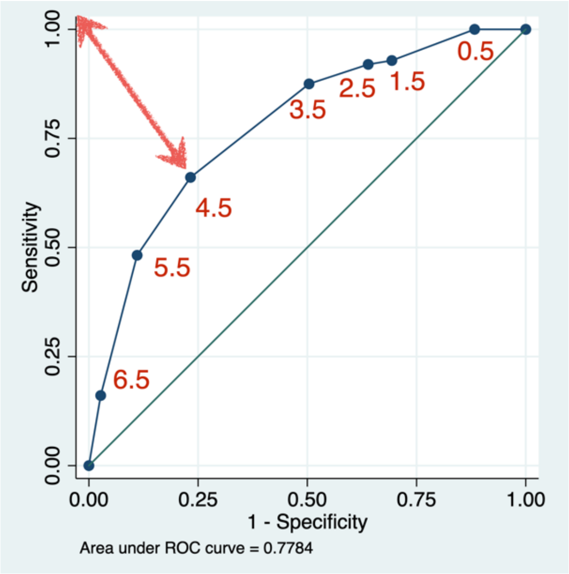

Sensitivity
0.00
0.25
0.50
0.75
1.00
1 - Specificity
0.00
0.25
0.50
0.75
1.00
0.5
1.5
2.5
3.5
4.5
5.5
6.5
Area under ROC curve = 0.7784
